# Genomic analysis and immune response in a murine mastitis model of vB_EcoM-UFV13, a potential biocontrol agent for use in dairy cows

**DOI:** 10.1038/s41598-018-24896-w

**Published:** 2018-05-01

**Authors:** Vinícius da Silva Duarte, Roberto Sousa Dias, Andrew M. Kropinski, Stefano Campanaro, Laura Treu, Carolina Siqueira, Marcella Silva Vieira, Isabela da Silva Paes, Gabriele Rocha Santana, Franciele Martins, Josicelli Souza Crispim, André da Silva Xavier, Camila Geovana Ferro, Pedro M. P. Vidigal, Cynthia Canêdo da Silva, Sérgio Oliveira de Paula

**Affiliations:** 1 Department of Microbiology, Federal University of Viçosa, Av. Peter Henry Rolfs, s/n, Campus Universitário, 36570-900 Viçosa, Minas Gerais Brazil; 2 Departments of Food Science, and Pathobiology, University of Guelph, Guelph, Ontario, N1G 2W1 Canada; 3 Department of Biology, University of Padova, Padova, Italy; 4 Department of Environmental Engineering, Technical University of Denmark, Miljoevej, Building 115, DK-2800 Kgs, Lyngby, Denmark; 5 Department of General Biology, Federal University of Viçosa, Av. Peter Henry Rolfs, s/n, Campus Universitário, 36570-900 Viçosa, Minas Gerais Brazil; 6 Embrapa Maize and Sorghum, Rodovia MG 424, Sete Lagoas, Minas Gerais Brazil; 7 Department of Plant Patology, Federal University of Viçosa, Av. Peter Henry Rolfs, s/n, Campus Universitário, 36570-900 Viçosa, Minas Gerais Brazil; 8 Núcleo de Análise de Biomoléculas (NuBioMol), Center of Biological Sciences, Federal University of Viçosa, Viçosa, Minas Gerais Brazil

## Abstract

Bovine mastitis remains the main cause of economic losses for dairy farmers. Mammary pathogenic *Escherichia coli* (MPEC) is related to an acute mastitis and its treatment is still based on the use of antibiotics. In the era of antimicrobial resistance (AMR), bacterial viruses (bacteriophages) present as an efficient treatment or prophylactic option. However, this makes it essential that its genetic structure, stability and interaction with the host immune system be thoroughly characterized. The present study analyzed a novel, broad host-range anti-mastitis agent, the *T4virus* vB_EcoM-UFV13 in genomic terms, and its activity against a MPEC strain in an experimental *E. coli*-induced mastitis mouse model. 4,975 Single Nucleotide Polymorphisms (SNPs) were assigned between vB_EcoM-UFV13 and *E. coli* phage T4 genomes with high impact on coding sequences (CDS) (37.60%) for virion proteins. Phylogenetic trees and genome analysis supported a recent infection mix between vB_EcoM-UFV13 and Shigella phage Shfl2. After a viral stability evaluation (e.g pH and temperature), intramammary administration (MOI 10) resulted in a 10-fold reduction in bacterial load. Furthermore, pro-inflammatory cytokines, such as IL-6 and TNF-α, were observed after viral treatment. This work brings the whole characterization and immune response to vB_EcoM-UFV13, a biocontrol candidate for bovine mastitis.

## Introduction

Bovine mastitis remains the main cause of economic losses for dairy farmers, estimated at $ (US)533 billion worldwide, as well as public health concerns, since low quality milk can be considered a vehicle for pathogen transmission^1–3^.

Mastitis treatment is typically based on the use of short and long-acting antibiotics, respectively, during the lactation and dry period^4^. In terms of the lactation period and specifically regarding clinical mastitis, *Escherichia coli*, *Streptococcus uberis*, *Streptococcus dysgalactiae* and *Staphylococcus aureus* are the main etiological agents involved that have been routinely isolated^5^. Among these pathogens, mammary pathogenic *Escherichia coli* (MPEC) is responsible for an acute mastitis characterized by inflammation, increased somatic cell count (SCC) and impaired milk quality even after the infection has been cured^6^.

Using *E. coli* strains obtained from different types of mastitis (*e.g* per-acute and persistent) and the non-pathogenic strain K71, Blum *et al*. (2017) performed a mammary immune response comparison in experimentally infected cows and noticed differences regarding TNF-α, IL-6 and IL-17 secretion levels for each MPEC.

Within the current scenario of widespread antibiotic-resistant bacteria, bacterial viruses present as an efficient therapeutic or prophylactic tool in order to control different pathogens in dairy cows at different lactation stages. This is supported by current *in vitro* and *in vivo* assays^7–13^.

Considered a model system in molecular biology, coliphage T4 has been studied since the 1940s and possesses about 300 genes organized in a 168.9 kb linear dsDNA with an average GC content of 34.5%^14^. Lytic viruses related to T4 have awoken interest for their application in phage therapy due the absence of lysogenic modules, a broad-host-range (from *Proteobacteria* to *Cyanobacteria* phyla) and, recently, the identification of virion-associated peptidoglycan hydrolases (VAPGHs), which are considered potential enzybiotics^15–17^.

Indeed, T4 was used by Bruttin^18^ in the first safety test of phage therapy in humans and in immunological assays in order to elucidate the interactions between viruses, its host and the immune system. Bocian *et al*.^19^ investigated how purified T4 phage and T4-generated *E. coli* lysate impact immune cells differentiation, highlighting that lysis of Gram-negative bacteria by phages might not trigger excessive monocyte induction.

Currently, several studies have been exploited the use of mouse models in the attempt to evaluate novels anti-mastitis drugs mainly against *E. coli* and *S. aureus*^20–26^. The use of animal models is time and cost effective approach, along with a previous step for pre-clinical and clinical assays^27^. However, the absence of intrinsic cow factors can be considered a bottleneck when data is analyzed^28^.

The aims of the present study were to characterize the *Escherichia* phage UFV13, a *T4virus*, in genomic, protein and physiological terms and evaluate the immune response in an experimental *E. coli*-induced mastitis mouse model with the aim to use it in clinical trials to control mastitis in dairy cows.

## Materials and Methods

### vB_EcoM-UFV13 isolation and purification

vB_EcoM-UFV13 (UFV13) was obtained from the sewerage system of Viçosa, Minas Gerais state, Brazil and was propagated on *Escherichia coli* 30 following the well-established Sambrook & Russell^29^ protocol. This virus belongs to the bacterial virus collection of the Laboratório de Imunovirologia Molecular (LIMV) at Universidade Federal de Viçosa (UFV), Viçosa city, Brazil.

After virus propagation, viral particles were purified by a three-step protocol using ion exchange and desalting columns in a chromatography system (ÄKTAprime plus, GE Healthcare Life Sciences, Uppsala, Sweden). Briefly, an initial step using HiTrap Desalting prepacked column (GE Healthcare Life Sciences, Uppsala, Sweden) was conducted to remove any salts used at virus propagation stages and the first two peaks were collected and purified using an ion exchange chromatography column, with specific fractions (7 to 11) driven to the final step, a new desalting process. For the anion exchange column, start (20 mM Tris-HC, pH 8.0) and elution buffers (20 mM Tris-HC, 1 M NaCl, pH 8.0) were used, whereas in desalting steps a phosphate buffer (20 mM sodium phosphate, 0.15 M NaCl, pH 7.0) was prepared. Flow rate of 5 mL.min^−1^ was adopted for both columns. Finally, viral titer was measured at 37 °C by double-agar overlay method^30^ using *E. coli* 30 as the plating host. Phage stocks were stored at 4 °C for further analysis.

### Bioinformatic analysis

Phage genome extraction, sequencing and annotation methodologies are described according to Duarte *et al*.^31^. Hypothetical proteins were manually checked for homologs using UNIPROT database. Protein isoelectric point and molecular weight were obtained using ExPASy^32^. Putative tRNAs and Rho-independent transcription terminators were, respectively, predicted using tRNAscan-SE^33^ and ARNnold web tool^34^. The Database of Gene Regulation in Bacteriophages (phiSITE)^35^ was used in order to check the three major classes of promoters (early, middle and late) as well as to confirm putative Rho-independent terminators. The CGView Server was used to generate a UFV13 genome graphical map^36^.

Nucleotide differences between UFV13 and Enterobacteria phage T4 (accession number AF158101.6) genomes were performed by checking reading alignments. High-quality Illumina reads were filtered and adaptor sequences were removed using Trimmomatic software (ver 0.33)^37^ (parameters: LEADING:10 TRAILING:10 SLIDINGWINDOW:4:15 MINLEN:65) and aligned to Enterobacteria phage T4 using Bowtie2 software (v2.2.4)^38^. SAMtools^39^ was used to convert the output SAM format to BAM format and, subsequently, to sort the BAM file. The sorted BAM file was processed with mpileup tool (SAMtools package) in order to extract the variants. The Binary Call Format (BCF) created was converted to VCF format using BCFtools^39^. VCF file and Enterobacteria phage T4 genes were used as input for SnpEff program^40^. Only variants with predicted “high” or “moderate” impact on the protein-coding gene were analyzed.

Whole-genomes that represent each genus from the subfamily *Tevenvirinae* (*T4virus*, *Cc31virus*, *S16virus*, *Js98virus* and *Sp18virus)* deposited on the International Committee on Taxonomy of Viruses (ICTV) were downloaded from NCBI (accession numbers are provided in Supplementary Table 4) and aligned with UFV13 genome using Progressive MAUVE^41^. Whole-genome single nucleotide polymorphisms (SNPs) were extracted as described in Treu *et al*.^42^ and a SNP-based phylogenetic tree was constructed using PHYLIP package^43^ and visualized by dendroscope^44^. Furthermore, a whole-genome phylogenetic tree was formulated aligning previously cited genomes from the *Tevenvirinae* subfamily. Online tools ClustalW2^45^ and MAFTTT version 7^46^ were used for multiple alignment. A neighbor-joining tree was drafted with MEGA7^47^ and visualized with FigTree (http://tree.bio.ed.ac.uk/software/figtree/).

*In silico* bacterial hosts of UFV13 were predicted using HostPhinder^48^.

### vB_EcoM-UFV13 structural protein analysis

With the aim of obtaining the UFV13 structural protein profile, proteomic analysis was conducted.

Viral propagation/purification (see item 2.2) were conducted and phage particles concentrated by adding NaCl 1 M and polyethylene glycol 8000 (PEG8000) 10% (w/v). The mixture was kept overnight at 4 °C. After a centrifugation (10,000 × *g*, 15 min), viral pellet was resuspended in 1 mL of SM buffer and one volume of chloroform followed by centrifugation at 4,000 *g* for 10 min. An equal volume of trichloroacetic acid (TCA) 10% (v/v) was added to the supernatant and incubated on ice for 30 min. The precipitated viral proteins were collected by centrifugation (11,000 × *g*, 20 min), washed three times with acetone (11,000 × *g*, 10 min) and resuspended in water. The *BCA Protein Assay Kit* (Boster Biological Technology, Wuhan, China) was used to estimate protein concentrations.

For protein profiling, the viral proteins were separated via sodium dodecyl sulfate-polyacrilamide electrophoresis (SDS-PAGE) on a15% gel^49^. Protein bands were highlighted by Coomassie Brilliant Blue R-250 dye and removed as described by Shevchenko *et al*.^50^. Mass spectrum (MS) was acquired by matrix-assisted laser desorption-ionization time of flight (MALDI/TOF-TOF) (Ultraflex III - BRUKER DALTONICS). Spectra intervals between 500 and 3,500 kDa were selected and forwarded for MS/MS analysis. Results were obtained by Mascot™ (Matriz Science) software using the NCBl nr protein database. Only proteins and peptides indicated as significant by Mascot were considered for further analysis.

### Physiological features

Physiological features were assessed in triplicate following the protocols described by Jurczak-Kurek *et al*.^51^. In each of the following cases, following treatment the phage preparation was diluted and tittered as described above (2.2).

#### pH stability

Viral capability at different acidic and alkaline pH values was evaluated. One mL of purified viruses were transferred to 9 mL LB medium with pH 2, pH 4, pH 7 (control), pH 10 and pH 12 at a 1:9 ratio and incubated for 1 h at 37 °C, being proceeded by a 10-fold serial dilution and plating as described in item 2.2. After overnight incubation at 37 °C, virus stability was determined by the percentage of viruses able to produce lysis plate.

#### Thermal stability

Thermal stability studies on LB-diluted phage suspensions were made at −20 °C, 40 °C, 62 °C and 95 °C, respectively, for 12 h, 40 min, 40 min and 5 min. After these periods, a serial 10-fold dilution was conducted and a specific aliquot was plated and incubated overnight at 37 °C. Viruses that did not undergo any thermal treatment were used as a control.

#### Viral ability to propagate in different temperatures

Viral replication at different temperatures (4 °C, 22 °C, 30 °C and 37 °C) was assessed by spot-assay after 24 hours of incubation using a 10-fold serially diluted viral stock in LB medium.

#### Osmotic shock effect

In order to verify the effect of the osmotic shock on virus particles, a stock aliquot was transferred to TM buffer (10 mM Tris–HCl, 10 mM MgSO_4_; pH 7.2) with sodium chloride 4.5 M, incubated at room temperature for 15 min and quickly diluted in TM buffer without sodium chloride. Bacterial viruses incubated in TM buffer without sodium chloride were used as a control.

#### Antiviral resistance

Antiviral activity of sodium dodecyl sulfate (SDS), sodium lauroyl sarcosinate (Sarkosyl) and cetyltrimethylammonium bromide (CTAB) on virus particles was determined incubating a standardized viral suspension with 0.09% SDS (20 min at 45 °C), 0.1% CTB (1 min at 22 °C) and 0.1% Sarkosyl (10 min at 22 °C). Controls were done considering the same conditions for each antiviral compound but they were substituted for TM buffer.

#### Organic solvent effect

To study the effect of four different organic solvents, a viral suspension was added to 63% ethanol, 90% acetone, 90% chloroform and 50% dimethyl sulfoxide (DMSO). Mixtures were incubated for 1 h at 22 °C (ethanol and acetone), 1.5 h at 4 °C (chloroform) and 10 min at 4 °C (DMSO). In the next step, 10-fold dilutions in TM buffer (10 mM Tris–HCl, 10 mM MgSO_4_; pH 7.2) were prepared and used for plating. Phages incubated in TM buffer under conditions described above, were used as a control.

### Animal model and immune response

#### Escherichia coli 30 strain

The mammary-pathogenic *E. coli* 30 was isolated from a dairy cow with acute mastitis and kindly provided by Brazilian Agricultural Research Corporation (Empresa Brasileira de Pesquisa Agropecuária – EMBRAPA) Dairy Cattle (Juiz de Fora, Minas Gerais, Brazil). *E. coli* 30 was evaluated for its ability to form biofilm as well as for motility capacity. Motility assays were conducted according to Deziel *et al*.^52^. Briefly, an overnight *E. coli* 30 aliquot was washed, suspended in distilled and sterilized water, and inoculated in King B medium (peptone 20 g/L, MgSO_4_.7H_2_O 1.5 g/L, K_2_HPO_4_ 1.5 g/L), supplemented with 1.5, 0.5 and 0.3% of agar for twitching, swarming and swimming tests, respectively. Biofilm assay followed the common crystal violet (CV) staining method^53^. After incubation (37 °C for 48 h), wells were washed 3 times with PBS buffer to remove not adherent cells. CV was added at a minimum volume capable overcome bacterial suspension volume, making possible quantify all bacterial biomass, and incubated for 30 minutes at room temperature. To biomass quantify, CV was removed, wells washed with PBS, added ethanol to solubilize biomass internal CV crystals, and optical density measured at 560 nm. All assays were performed in triplicate.

Antimicrobial susceptibility test of *E. coli* 30 was assessed by disc diffusion assay using polisensidiscs for 25 different antibiotics (DME, Araçatuba, São Paulo, Brazil) following the manufacture’s recommendation. The results were interpreted according to the standards of the Clinical and Laboratory Standards Institute (CLSI)^54^.

#### *E. coli*-induced mastitis in mouse

With the aim to evaluate UFV13 effectiveness and the immune response against *E. coli* 30 *in vivo*, an experimental *E. coli*-induced mastitis mouse model was used. The trial followed the methodology described by Chandler^55^, with some modifications, and was approved by the Ethics Committee (Comissão de ética no uso de animais/UFV) according to the protocol 64/2016. Lactating Balb/c female mice (5 to 15 days) was intraperitoneally anesthetized with 10% Ketamine and 2% Xylazine, with subsequent surgical operation of the mammary gland by cutting the teat canal of the last two abdominal ceilings (R5 and L5 of each animal were used) (Supplementary Figure 4). *E. coli* 30 (100 UFC/ml), PBS and phage served as the control groups, while phage plus bacteria was considered the treatment group (MOI 10). Viral addition was done four hours after bacterial inoculation. Three animals were used to perform each experimental group.

In order to assess the lytic activity of the phage vB_EcoM-UFV13 in mammary glands, animals were euthanized 48 hours after the treatment by anesthetic overdose. Infected mammary glands were removed and transferred to 1.5 ml of (PBS), macerated and serially diluted (1:10) in PBS buffer. A microdrop assay^56^ was performed to estimate *E. coli 30* colony-forming unit (CFU).

Cytokines IL-6, TNF-α, IL-2, IFN-γ, IL-4, IL-10 and IL-17A obtained from macerated mammary gland (L5 and R5 from each animal was pooled) were simultaneously quantified by the Cytometric Bead Array kit (CBA, BD Bioscience) in a BD FACSVerse Flow cytometry following manufacturer’s recommendations.

### Histological analysis

For histology, mice were euthanized 48 hours after phage treatment. The mammary glands were removed and fixed in Karnovsky fixative (paraformaldehyde 4% and glutaraldehyde 4%, pH 7.3). Further, the tissues were embedded in paraffin and a 5 µm section was obtained by microtomy, stained with hematoxylin and eosin (H & E) and observed under light microscopy. The images were acquired under an Olympus DP73 microscope.

### Statistical analysis

Statistical analysis was performed with GraphPad Instat 3 software (GraphPad, La Jolla, CA, USA) using the one-way analysis of variance (ANOVA) at 95% accuracy level to evaluate the differences between mammary gland cytokine production under different conditions. This assay was set up in triplicate and, for each animal, the two abdominal ceilings were pooled into one sample. Tukey’s test was used as *post hoc* test.

## Results and Discussion

### vB_EcoM-UFV13 genome analysis

Bacteriophage UFV13 was isolated from samples obtained in the sewage system of Viçosa, Minas Gerais, Brazil, a well-known source of novel viruses^57^.

**Figure 1:**
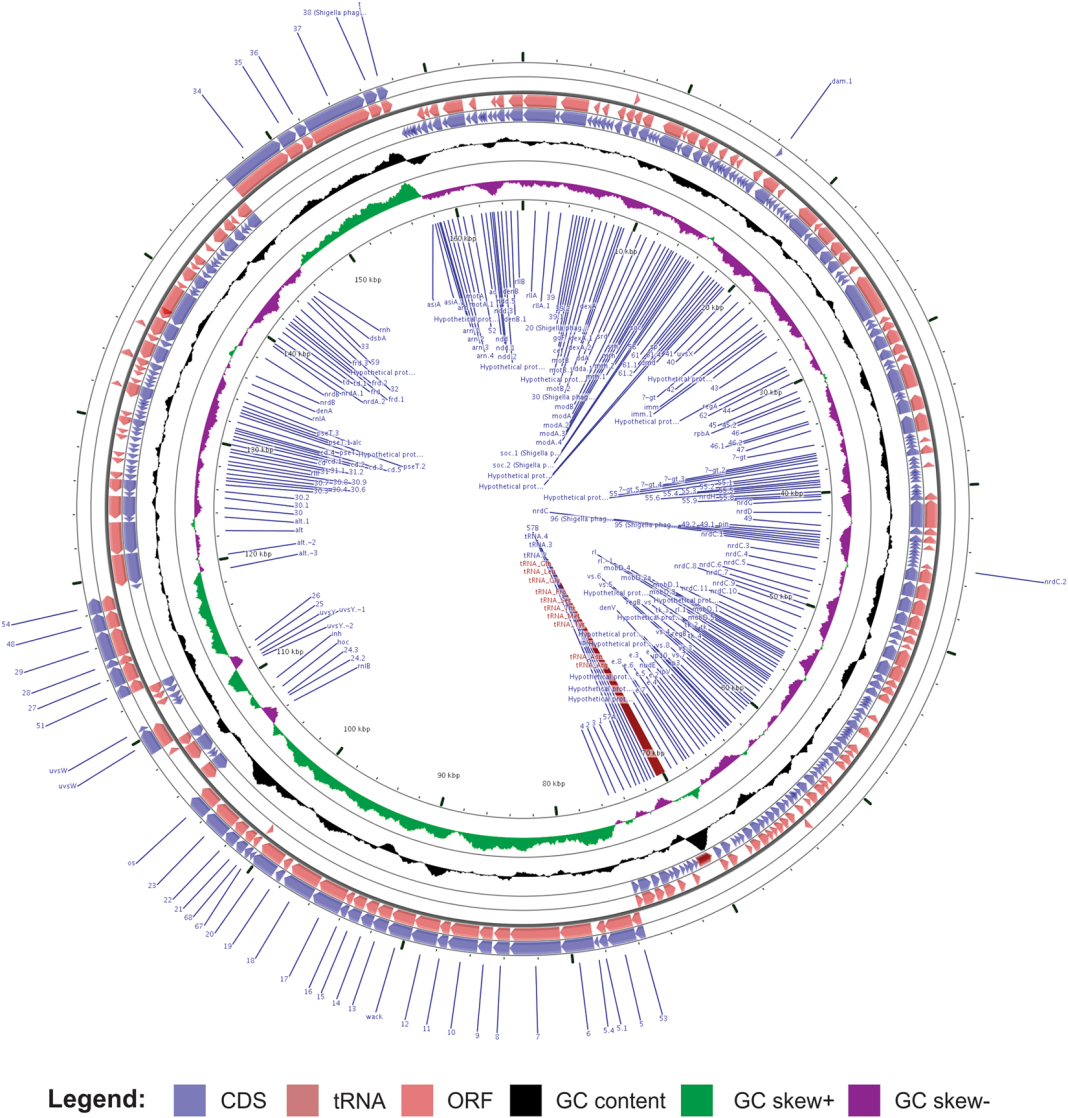
Genome map of vB_EcoM-UFV13. The linear genome was circularized in order to improve its visualization. CDS, ORF, GC content, GC skew+ and GC skew- are reported in circles from outside inwards.

From the genome sequence (165,772 bp, GC content 34.8%), 269 ORFs were predicted and annotated. The size is similar to that of other members of the T4virus genus^58^. In addition, a total of ten tRNA encoding genes (Gln, Leu, Gly, Pro, Ser, Thr, Met, Tyr, Asn and Arg) were identified and are organized in a gene cluster without introns or pseudogenes (Fig. 1). 13 ORFs showed an identity below 70% with the reference phage T4, while 24 ORFs were annotated as hypothetical protein coding sequences. ORFs 50 and 232 are, respectively, coding sequences for T2 and T6 bacteriophage proteins. 13 ORFs encode for Shigella phage proteins (pSs-1, Shfl2, Shf125875, SH7, SHBML-50-1). Genes for IpII and IpX were not identified in the UFV13 phage genome. Gene and protein information such as genomic coordinates, protein weight, pI and putative function for each UFV13 ORF are reported in Supplementary Table 1.

Functional categorization of UFV13 genes (Supplementary Table 2) revealed that nonessential and auxiliary genes mainly related to homing endonucleases of introns such as *I-TevI*, *I-TevII* and *I-TevIII* are absent, as with some of their related introns like *mob* and *seg* genes (Table 1). Homing endonucleases of introns are considered systems associated with gene conversion events, transference of mobile elements and gene exclusion in mixed infections^14^. In fact, genome analysis suggests a recent mixed infection among UFV13 and Shigella phage Shfl2, once *segF* was substituted by *soc.1* and *soc.2* from Shigella phage Shfl2. According to Belle *et al*.^58^, *segF* was absent in T2 phages, but the region is occupied by *soc.1* and *soc.2*.

**Table-1:** Genes not predicted in vB_EcoM-UFV13 based on the reference genome Enterobacteria phage T4 (accession number NC_000866).

Gene	Function	Relevance
*mobA*	Pseudogene of Mob site-specific DNA endonuclease	Nonessential
*mobB*	Putative site-specific intron-like DNA endonuclease	Nonessential
*mobC*	Putative intron-like DNA endonuclease	Auxiliary
*mobD*	Putative site-specific DNA endonuclease	Nonessential
*mobE*	Putative mobile endonuclease	Nonessential
*segA*	Site-specific intron-like DNA endonuclease	Nonessential
*segB*	Probable site-specific intron-like DNA endonuclease	Nonessential
*segC*	Site-specific intron-like DNA endonuclease	Nonessential
*segD*	Probable site-specific intron-like DNA endonuclease	Nonessential
*segE*	Probable site-specific intron-like DNA endonuclease	Nonessential
*segF*	Intron-like endonuclease. A probable fusion protein, generated from 56 and 69 by hopping of ribosomes across a pseudoknot, is larger	Nonessential
*repEA*	Protein auxiliary for initiation from *oriE*	Auxiliary
*repEB*	Protein required for initiation from *oriE*	Auxiliary
*I-TevI*	Intron-homing endonuclease	Nonessential
*I-TevII*	Endonuclease for nrdD-intron homing	Nonessential
*I-TevIII*	Defective intron homing endonuclease	Nonessential
*rnaD*	Stable RNA	Auxiliary
*stp*	Peptide modulating host restriction system	Auxiliary
*nrdA*	Ribonucleotide reductase α subunit	Auxiliary; *nrd*-defective hosts

Function and relevance were integrally withdrawn from Miller *et al*. (2003).

Similarity analysis using the deposited genomes from Yersinia phage PST and Shigella phage Shfl2 showed that the bacteriophage UFV13 has 97% identity with both phages. However, it has a larger percentage of aligned genome (96%) for the Shfl2, and 90% of genome aligned to phage PST (Supplementary Figure 1). Comeau *et al*.^59^, characterized Yersinia phage PST genome, containing dsDNA with 167,785 bp, 35.3% GC content and 9 tRNAs, whose values are near to those found for the phage UFV13, with 34.8% GC content. Jun *et al*., (2016) identified 10 tRNAs for both pSs-1 and Shfl2 viruses infecting *Shigella* spp. as host. Despite little existing knowledge about tRNAs functioning in phage life cycles, high tRNAs numbers found in some bacteriophages could be proportional to phage genomes sizes and are related to a short latent period and high burst size value^60^.

In the total, 35, 21 and 31, early, middle and late promoter regions respectively, were predicted, which corresponds to 73% of the promoters identified for the T4 genome (Supplementary Table 3). As expected, no promoter regions for *mob* and *seg* genes were found, along with *I-TevII* and internal protein II (*ipII*).

Overall, termination of transcription in T4 bacteriophages occurs by an intrinsic termination signal, a stem-loop arrangement accompanied by a U-rich region, or a Rho-dependent termination mechanism^61^. For UFV13, 82 Rho-independent transcription terminators were predicted and compared against phiSITE database. 30 sequences displayed high identity with the reference phage, while five were not predicted but were found using the sequences available on the phiSITE. Terminators for the genes *45*, *5.4*/*6′*, *34*/*35*, *35*, *37*, *nrdA*, *uvsY.−2* and 56/*segF* were not found or even identified using phiSITE (Table 2), while for the genes such as *regA*, *wac*, *24(b)* and *30.9(b)* were, respectively, detected on the ORFs 52, 163, 178 and 208. According to Miller *et al*. (2003), generally 34 terminators are found in the T4 genome. The absence of terminators for the genes *nrdA* and *56/segF* can be explained by their absence, since these genes were not annotated. The significance of terminators within genes is unknown but have been described for T4 phages^14^.

**Table-2:** Main features of the predicted Rho-independent transcription terminators.

Intergenic location	Genome position	Strand	Predicted Rho-independent transcription terminator site	Free energy (kcal/mol)
39.1	4310..4341	Minus	TTTAAATAAAAGGCCTTCGGGCCTTTAGCTTTATG	−10.60
soc	15135..15168	Minus	AATTCAAGGACTCCTTCGGGAGTCCTTTTTCATT	−16.30
uvsX-40	21337..21371	*	*	*
Unknown gene	25445..25477	Minus	TAAATCTAGGGACCTCCGGGTCCCTTTTTCACAC	−12.10
regA	28228..28259	*	*	*
α-gt	35109..35140	Minus	ACAAAATAAAGGGCTTCGGCCCTTTAGCTTTATA	−10.60
α-gt.2	36378..36411	Minus	TATGCGGATAGGAGCTTCGGCTCCTATATTGCTT	−14.20
55.3	38744..38775	Minus	GTTTAGCTAAGGGCTTCGGCCCTTTTTGGATAAT	−10.60
nrdH	39707..39739	Minus	GATTAAGACGGGCCCTCTGGGCCTTTCTTTCTCG	−8.80
Pin	43124..43166	Minus	AAATACCCTTATCTATTTAAGGTAAGGGTTTATTA	−10.70
nrdC.11	51781..51818	Minus	AATGATAGGGAGCCTTCGGGCTCCCTTTTTTATT	−18.40
rI.−1	55358..55389	Minus	TAACATTAGTCTCCTTCGGGAGACTTTTTTCATT	−13.50
Vs	58098..58128	Minus	TATATCAAGGGCGATATTGTCGCCCTTTTTCTTTA	−11.40
e.6	66465..66498	Minus	ATAATGATAAGGGGCTTCGGCCCCTATTACTTGG	−13.90
RNA C	69462..69503	Minus	GCTTAGCCCCAGCCGAAAGGTTGGGGCTTTTTA	−17.40
8	84691..84728	Plus	TAAATTAAGGGAGCCCATGGGCTCCCTTTTTCTT	−16.50
wac	91126..91161	*	*	*
19	98662..98695	Minus	AAGCAGGATGGGGATTTCTCCCCATTCaTTTTAT	−14.50
23	151432..151461	Plus	AATTGAGGGAGCCTTCGGGTTCCCTTTTTCTTTA	−16.70
24(a)	105412..105447	Plus	AAAACAAAGGGACCTTTCGGTCCCTTTTTATTTA	−12.30
24(b)	105467..105499	*	*	*
hoc	106999..107032	Minus	TAATCATAAGGGGCTTCGGCCCCTTTCTTCATTT	−14.50
54	118931..118972	Plus	CTAACAATGGGGACCGAAAGGTCCCCATATTTTT	−19.90
alt.1	123501..123532	Minus	GATTACTAAAGGCCTTCGGGCCTTTAaTTTTATAA	−14.80
30.9(a)	128117..128154	Minus	AAGTTGAGGACTCCTTCGGGAGTCCTTTTTTATT	−16.30
30.9(b)	128162..128198	*	*	*
nrdB	135904..135939	Minus	TTAAGGAGTGGGCCGCAAGGCCCATTTTATTATG	−15.30
32	143116..143152	Minus	ATTAATTGGGGACCTCTAGGGTCCCCTTTTTTAT	−14.90
T	157574..157618	Plus	CAAACCCTCGTTGAATTCGTCGATGAGGGTTTTC	−11.10
motA.1	160357..160396	Minus	ATTTTAGGGAGAGCTTCGGCTCTCCCTTTTTTAT	−19.60
Ac	161968..162004	Minus	TGCCCTTGCTACTTTATTGGTAGCAcTATATTATG	−8.60
denB.1	164573..164601	Minus	CAAATAAATAAGGGCTTCGGCCCTTTTGTTTTAA	−10.60
5.4	78461..78499	Minus	GTCACTCCGCCATGTGTTTCATATGGCTTTTTAA	−10.20
Stp	162165..162200	Plus	TTCCTCACTGGCGTCCGAAGACGCCTTTAATTTT	−10.30
rIIB	164798..164834	Plus	TCCTTAGTTAAGGGCCGAAGCCCTTATTTAAATT	−10.00

The asterisk means that absence of terminator prediction by using Arnold or phiSITE programs.

**Figure 2:**
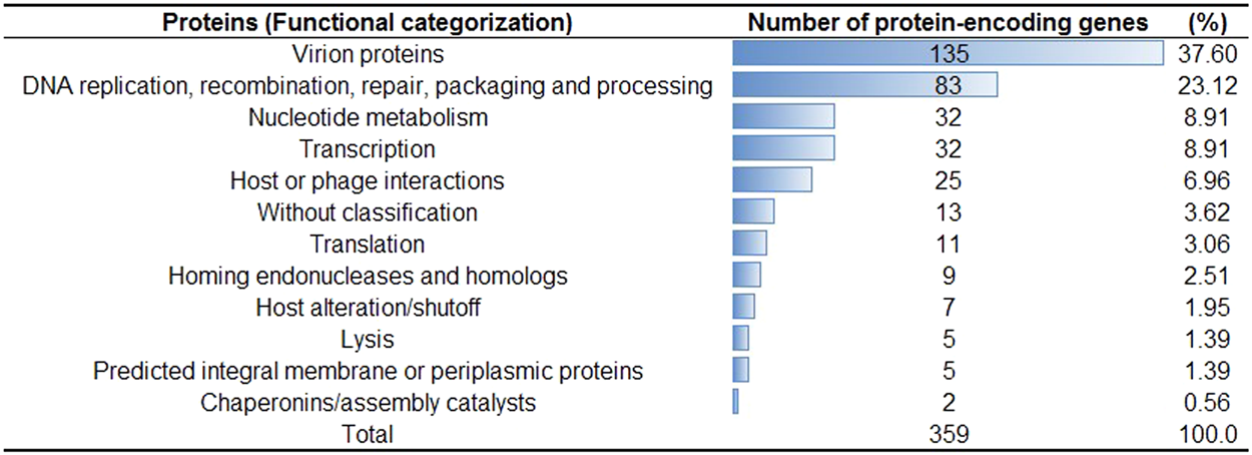
Variants calling between vB_EcoM-UFV13 and Enterobacteria phage T4 were predicted using SnpEff. Only variants with predicted “high” or “moderate” impact on the protein-coding gene were analyzed and functionally categorized.

A total of 5,071 filtered variants (4,975 SNPs, 86 insertions and 10 deletions) were identified (1 variant every 33 bases) between UFV13 and T4. 83,656 effects were assigned and categorized (395 high (0.472%), 1,670 low (1.996%), 2,722 moderate (3.25%) and 78,869 modifier (94.28%). The number of effects by functional class is: missense 2,879 (60.79%), nonsense 187 (3.95%) and silent 1,670 (35.26%). Considering only genes with high or moderate variants, the mainly affected protein category was that related to virion structural proteins, with gp7 being the most affected (Fig. 2). This protein is considered the second largest protein in the baseplate and is one of the seven components associated with wedge assembly and stability^62^. The large number of SNPs found in virion proteins reflects the diversification of phage UFV13 from the classical T4 and can be associated with the high capability to infect different bacterial genus such as *Escherichia*, *Morganella* and *Shigella*, an uncommon phage feature (data not shown).

**Figure 3:**
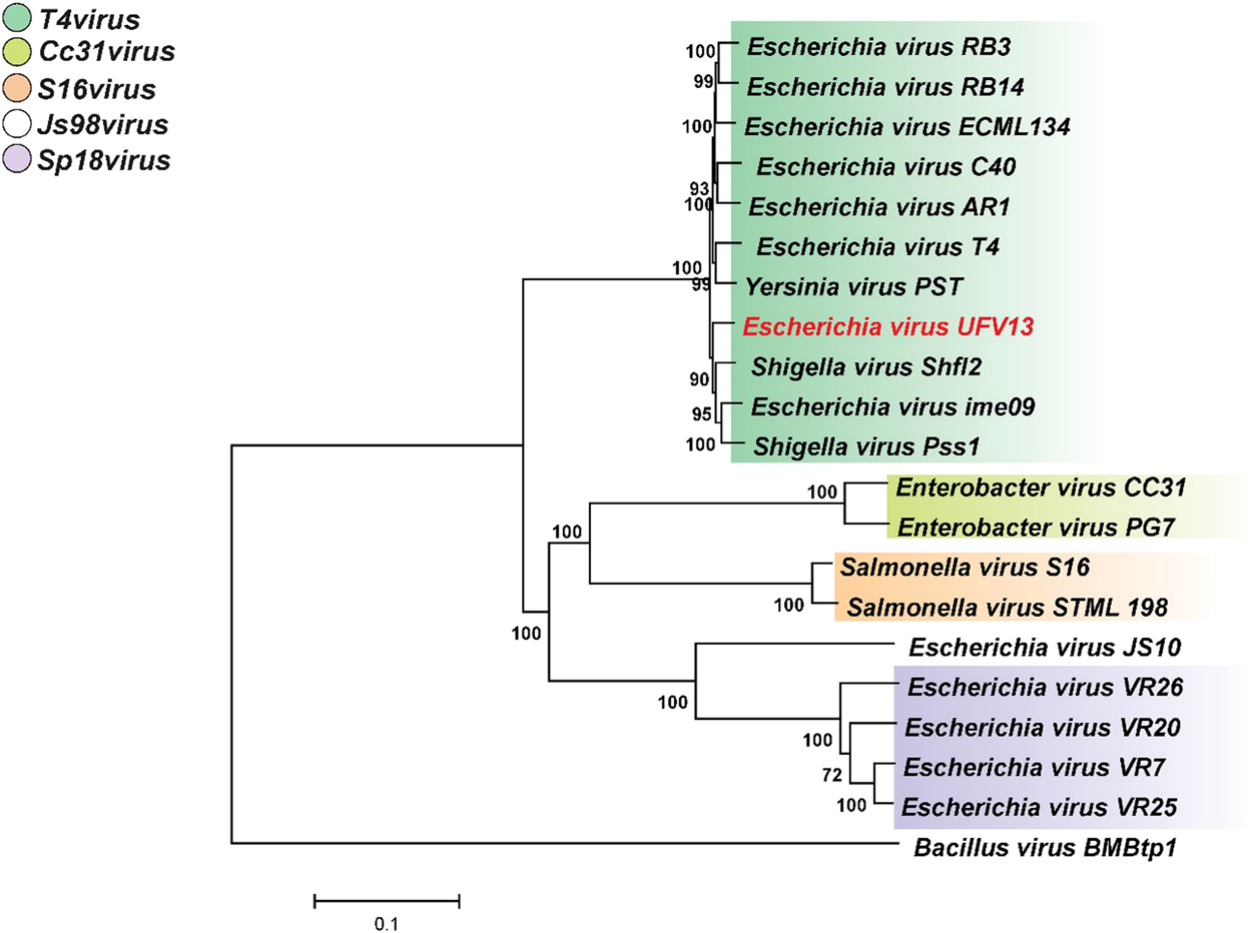
Phylogenetic relationship between phage UFV13 and genera belonging to the subfamily *Tevenvirinae* (*T4virus*, *Cc31virus*, *S16virus*, *Js98virus* and *Sp18virus*). vB_EcoM-UFV13 is most closely related to *Shigella* phage Shfl2 and *Yersinia* phage PST, and is clearly a member of the *T4virus* genus.

In order to evaluate the phylogenetic relationship between UFV13 and genera belonging to the subfamily *Tevenvirinae* (*T4virus*, *Cc31virus*, *S16virus*, *Js98virus* and *Sp18virus*), a whole-genome tree was constructed. The phylogenetic tree divided phages into four clusters with vB_EcoM-UFV13 possessing the closest relationship with Shigella Shfl2 and Yersinia phage PST (Fig. 3), a result also supported by the SNP-based phylogenetic tree (Supplementary Figure 2).

With the aim to establish the host range of phage UFV13 host range, *in silico* Host Phinder test was conducted. Four bacterial genera were predicted to be infected by UFV13: *Escherichia* (E-value: 5.9e^−1^), *Yersinia* (E-value: 5.8e^−1^), *Shigella* (E-value: 6.3e^−1^) and *Salmonella* (E-value: 1.1e^−2^).

### Viral protein analysis

SDS PAGE analysis revealed the presence of eight proteins (Supplementary Figure 3). Among these, four proteins were chosen and analyzed by MALDI/TOF-TOF, with the following being identified: UFV13_gp243 long tail fiber proximal subunit (139.95 kDa), *E. coli* chaperonin GroL (57.36 kDa), Escherichia phage *vB_EcoM_112* major capsid protein (56.09 KDa) and *E. coli* outer membrane protein C (36.77 kDa). These proteins are deposited at UNIPROT under accession numbers A0A160CBJ3, A0A017I9Q1, A0A160CBB0 and A0A148HSV3, correspondingly. The presence of a common viral receptor (OmpC) and the chaperonin GroL highlights the need for viral purification enhancement. Viral isoelectric points are usually below six and some of them are able to bind to anion and cation exchange matrixes. However, host cell DNA is efficiently removed by cation exchanger columns, while host cell proteins are effectively eliminated by anion exchange^63^.

### Physiological features

Viral ability to survive in a wide range of adverse conditions is a desired characteristic for therapeutic as well as a biological control agents^64^. Thus, phage stability was evaluated in different physical and chemical conditions.

**Figure 4:**
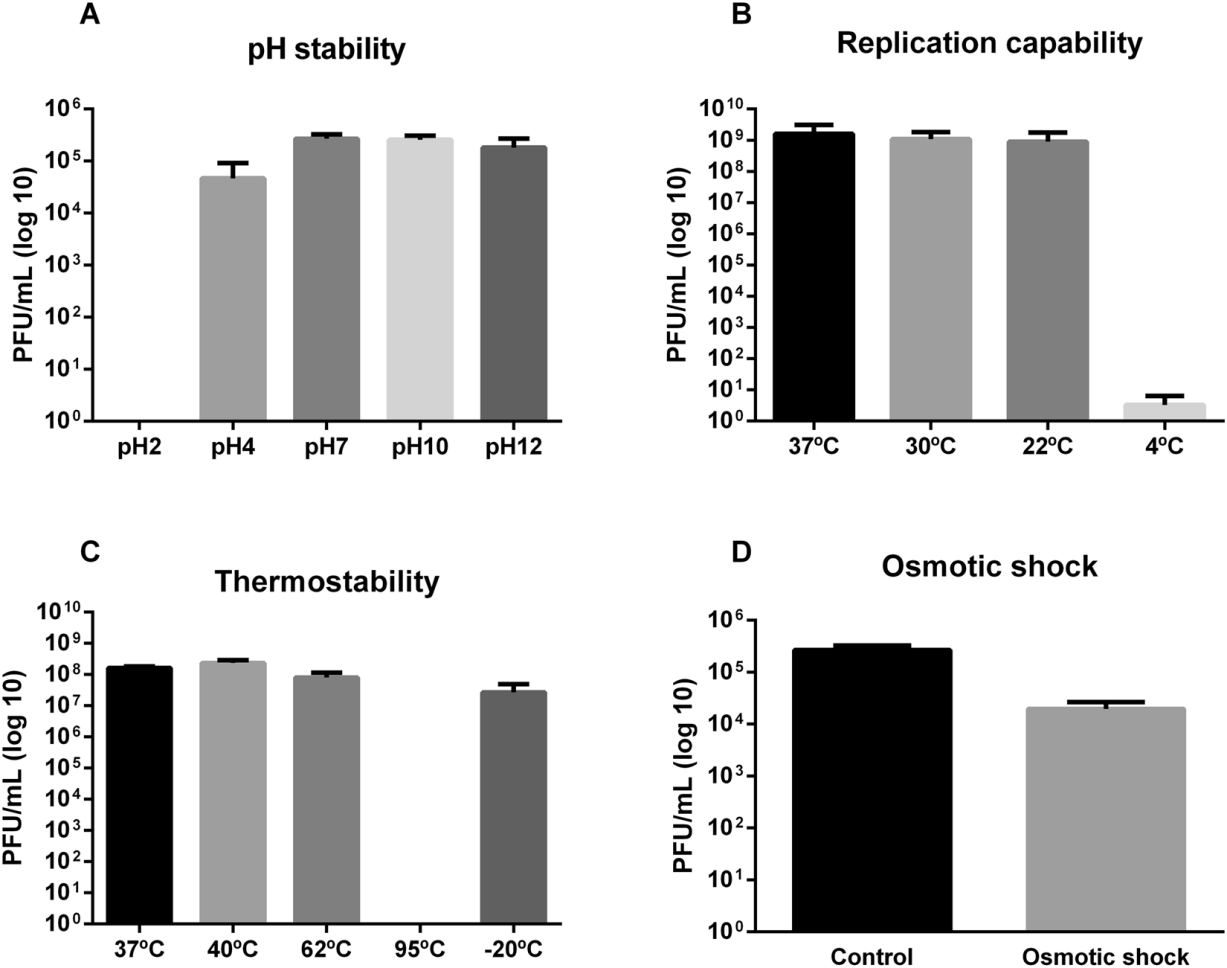
The stability of vB_EcoM-UFV13 under different conditions was evaluated. (**A**) Reductions of 100, 82, 4 and 32% of viable particles were observed after incubations, respectively, at pHs 2, 4, 10 and 12. (**B**) vB_EcoM-UFV13 was able to replicate at 30 and 22 °C with an efficiency of plating of 69 and 56%, corresponding; (**C**) vB_EcoM-UFV13 was inactivated at 95 °C for 5 min; (**D**) Osmotic shock changing reduced in 84% viral viability.

UFV13 was relatively stable within a pH range of 7.0–12.0. An approximate 1 log-fold reduction on viral titer was observed after incubation at pH 4.0 (18% of survivability) (Fig. 4A). Interestingly, phage UFV13 was inactivated after 1 h of incubation at pH 2.0, but not at pH 12 (68% viral viability), which is indicative of considerable virion stability at basic pH values.

UFV13 was able to lyse *E. coli* at 30 °C and 22 °C with a plating efficiency of 69 and 56%, respectively (Fig. 4B). No lysis plates were observed after storage at 4 °C, which may be indicative of DNA injection without host lysis.

Viral thermal inactivation occurred in the extreme tested temperatures 95 and −20 °C (0 and 24% survival, respectively), while viral titer dropped to 50% when UFV13 was incubated for 40 min at 62 °C (Fig. 4C). No significant reduction of phage titer was observed after thermal treatment at 40 °C.

**Figure 5:**
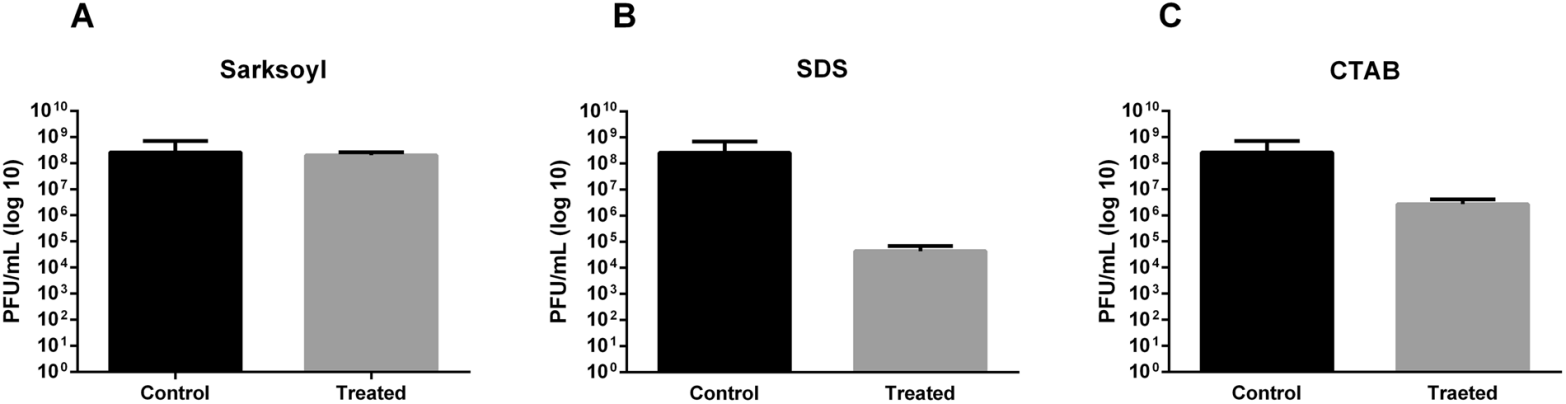
Viral stability under anionic and cationic detergents showed that vB_EcoM-UFV13 was sensible in all conditions.

It was found that osmotic pressure change, detergent and organic solvents determined a significant titer drop of UFV13. A survivability of 12% was observed when UFV13 underwent a rapid dilution from high-concentration NaCl buffer to low-concentration ones (Fig. 4D). The anionic detergent Sarkosyl reduced the number of viable viral particles by 74%, while CTAB and SDS resulted in a complete inactivation of this virus (Fig. 5). Anti-phage activity was also evidenced after exposure to ethanol, chloroform, acetone and DMSO.

Physiological features evaluated in this study are in accordance with Jurczak-Kurek *et al*. (2016). In a broad physiological study using 83 bacteriophages isolated from urban sewage, the same source of UFV13, it was verified that the vast majority of phages were sensitive to a temperature of 62 °C (survival below 70%), were able to survive in basic pH (ranging from 10 to 12), were susceptible to detergents and organic solvents, except chloroform, and were also resistant to osmotic shock.

Viral stability assays have also been shown that UFV13 can survive in raw milk and it has potential capability to survive in mastitic milk. In general, mastitis can dampen the quality of raw milk composition, which includes increased levels of Na^+^/Cl^−^ and pH^65^. Evaluating a bacteriophage cocktail composed of two T4 phages in raw milk against *E. coli*, Porter *et al*. (2016) observed a 3.3- to 5.6-log reduction of bacterial growth over a 12-h physiologic temperature. As discussed below (item 3.4), this study showed UFV13 activity against *E. coli* in lactating female mice.

### *E. coli*-induced mastitis mouse model

*E. coli* 30, an isolate obtained from a dairy cow with acute mastitis, displays resistance to 14 of 25 evaluated antibiotics (Supplementary Table 5). This bacterium is resistant to at least three different antimicrobial drug classes, which allows its classification as a multi-drug resistant strain^66^ Moreover, of the four types of virulence factors analyzed (biofilm forming capability and motility types: swarming, swimming and twitching) the *E. coli* 30 was positive for biofilm formation, swarming, and swimming, being negative only for the twitching (Supplementary Table 6). Studies correlated this virulence factors to an increase in pathogenicity and immune response evasion^67,68^.

Nowadays, several studies have been used mouse mastitis models with the aim to evaluate potential anti-mastitis agents for use in dairy cows^20–25,69–71^ although the authors are aware that cow factors are relevant in the mastitis establishment^28^.

In order to evaluate UFV13′s *in vivo* activity against an MPEC strain and immune response to this treatment, an *E. coli*-induced mastitis model was employed. A 10-fold reduction of bacterial load was observed after viral inoculation using MOI 10 (Supplementary Figure 5).

**Figure 6:**
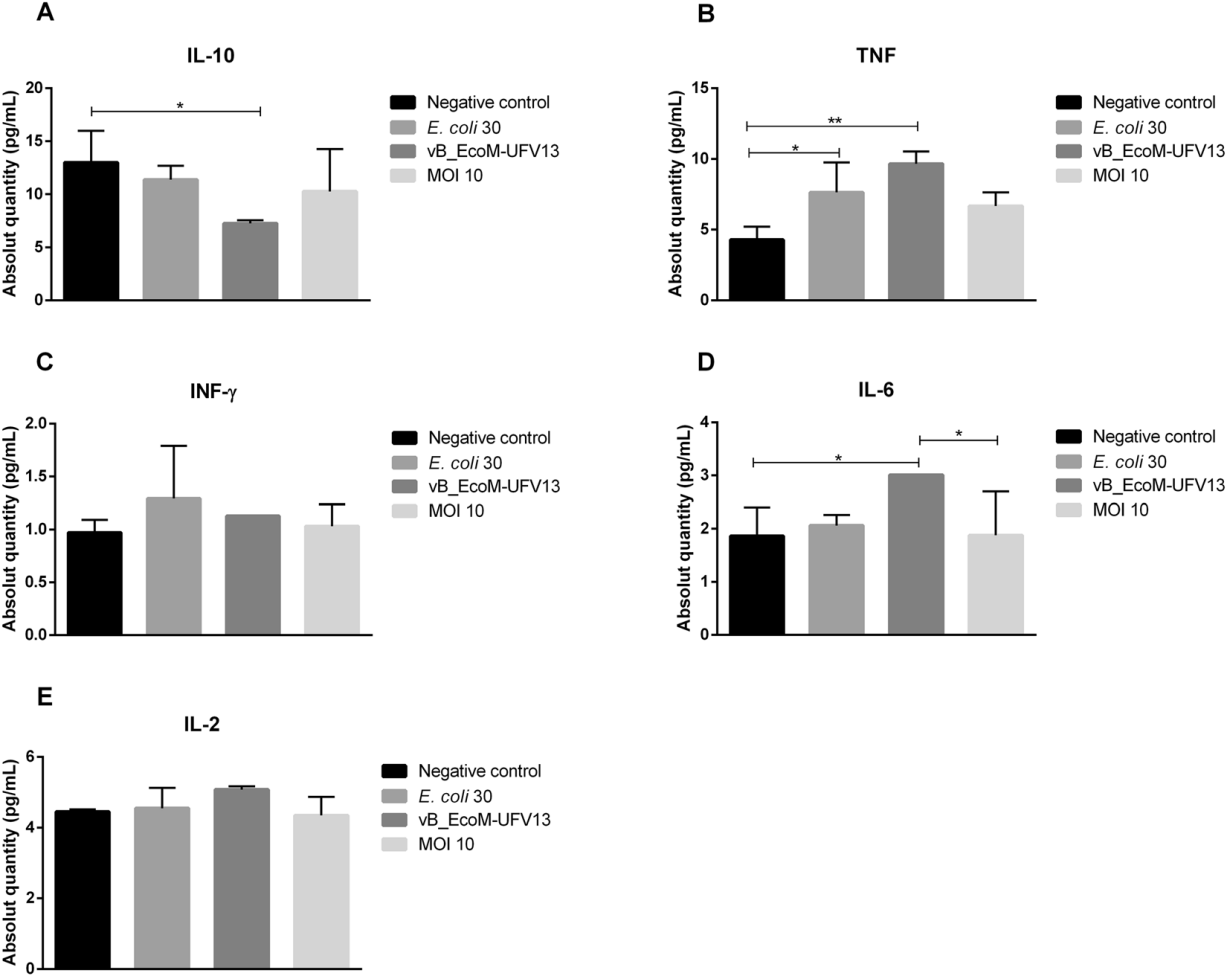
Five different cytokines (IL-6, TNF-α, IL-2, IFN-γ and IL-10) were locally measured. Only IL-10, TNF-α and IL-6 were statistically significant (*p < 0.05; **p < 0.01) and is indicative of a pro-inflammatory pattern after phage treatment. IL-17A and IL-4 levels were not detected by the Cytometric Bead Array kit.

Seven different cytokines (IL-6, TNF-α, IL-2, IFN-γ, IL-4, IL-10 and IL-17A) were measured, however only IL-10, TNF-α and IL-6 were identified as statistically significant (*p* < 0.05) (Fig. 6).

Studying whether T4 bacteriophage and T4-generated *E. coli* lysate influence cultures of peripheral blood mononuclear cells (PBMCs) activated or not by lipopolysaccharide (LPS), Bocian *et al*. (2016) observed that both preparations considerably increased the percentage of CD14+CD16−CD40+ and CD14+CD16−CD80 + monocytes in LPS-unactivated PBMCs cultures, as well as the concentration of IL-6 and IL-12. Notwithstanding, this result suggests that T4 bacteriophages may act both as a pro-inflammatory inducer, and also as CD40 activator, for this reason the increased expression of IL-6, IL-10, and TNF-α as a consequence of the presence of contaminating LPS left after the purification step rather than a property of the virion.

In an *In vivo* assessment of cytokine patterns followed by a single-dose T4 bacteriophage application in an *E. coli* induced mastitis mouse model, our work indicates that IL-10 levels decreased locally in phage therapeutic group when compared to the negative control (PBS buffer) (Fig. 6A). IL-10 has been extensively studied due its immunosuppressive features associated with the downregulation of pro-inflammatory cytokines, such as TNF-α and IFN-γ, and the resolution of the inflammatory process^72,73^. Evaluation of cytokine expression in the mammary gland in a mouse model of *Streptococcus agalactiae* mastitis performed by Trigo *et al*. (2009) revealed that the maximum concentration of IL-10 occurred after 72 hours and was correlated with a decreased level of TNF-α. This finding is in accordance with our results. Reduced IL-10 levels (Fig. 6A) and increased TNF- α (Fig. 6B) and IL-6 (Fig. 6D) is indicative of an ongoing inflammatory process. In fact, when only *E. coli* 30 was inoculated, an increased abundance of TNF-α was also observed when compared with the negative control. Although phage and *E. coli* 30 were individually able to induce an inflammatory response, phage treatment (10^3^ PFU) did not provoke an additive effect on the production of any pro-inflammatory cytokine. Indeed, the IL-6 level diminished (Fig. 6D) after phage treatment. Different to what was found by Trigo *et al*. (2009), IFN-γ levels were detected in all groups, however no statistical difference was observed between the groups, which was also applicable to IL-2 (Fig. 6C–E, respectively).

The absence of detectable IL-4 and IL-17A cytokines, respectively present in Th2 and Th17 inflammatory responses, indicates that the use of T4 phage in mammary glands infected with *E. coli* induces Th1 T-cell responses. Th1 pattern is involved in a cellular immune response that protects against intracellular infections by viruses and microorganisms that grow in macrophages^74^.

**Figure 7:**
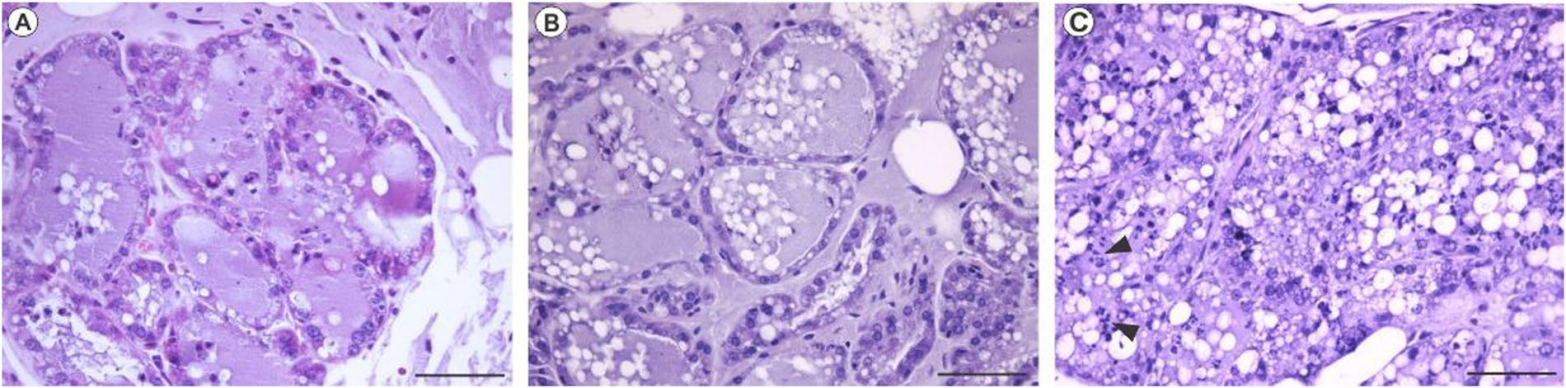
Histological analysis of mammary gland after PBS (**A**), *E. coli* 30 (**B**) and treatment with vB_EcoM-UFV13 (**C**). In C, neutrophil infiltration (indicated by arrow head) was detected 48 hours after phage treatment using MOI 10. Bars: 100 micrometers.

Histological analysis of the mammary gland in the control group (Fig. 7A) shows intact tissue morphology described by well delimited cells and acini, as well as the presence of fatty cells and a milky secretion, even when *E. coli* 30 (~100 cells) were inoculated (Fig. 7B). Inoculation of the phage UFV13 (MOI 10) in the treated group led to an inflammatory reaction characterized by tissue damage, neutrophil infiltrates and mischaracterization of the acini border, although low levels of pro-inflammatory cytokines have been found as discussed previously (Fig. 7C).

## Conclusion

Viral genomic analysis is a crucial step in bacteriophage screening for their use as an antibacterial agent. The UFV13 virus has no lysogenic modules or genes conferring antibiotic resistance. Viral stability analysis in the presence of detergents and organic solvents and incubation at different pHs and temperatures, have shown that the UFV13 virus presents high survivability at basic pHs and is relatively resistant under incubation at 62 °C for 40 min. vB_EcoM-UFV13 used in an animal model for mastitis reduced the total bacterial load by 90%, as well as inducing pro-inflammatory cytokines such as IL-6 and TNF-α, which makes it a potential biological agent capable of controlling acute infections caused by *E. coli* dairy cows.

## Electronic supplementary material

Supplementary Information
