# Supplementary material for: Genomic analysis and immune response in a murine mastitis model of vB_EcoM-UFV13, a potential biocontrol agent for use in dairy cows
Source: Sci Rep. 2018 May 1;8:6845. doi: 10.1038/s41598-018-24896-w (PMC5931544; doi:10.1038/s41598-018-24896-w)
Supplement: Supplementary file 1 — Supplementary Information [file 41598_2018_24896_MOESM1_ESM.doc]

# Supplementary Material

# Title: Genomic analysis and immune response in a murine mastitis model of vB_EcoM-UFV13, a potential biocontrol agent for use in dairy cows.

**Authors:**

Vinícius da Silva Duarte^a^, Roberto Sousa Dias^a^, Andrew M. Kropinski^b^, Stefano Campanaro^c^, Laura Treu^c, d^, Carolina Siqueira^e^, Marcella Silva Vieira^e^, Isabela da Silva Paes^e^, Gabriele Rocha Santana^e^, Franciele Martins^e^, Josicelli Souza Crispim^a^, André da Silva Xavier^f^, Camila Geovana Ferro^g^, Pedro M. P. Vidigal^h^, Cynthia Canêdo da Silva^a^, Sérgio Oliveira de Paula^e^

**Affiliations:**

^a^ Department of Microbiology, Federal University of Viçosa, Av. Peter Henry Rolfs, s/n, Campus Universitário, 36570-900, Viçosa, Minas Gerais, Brazil

^b^ Departments of Food Science, and Pathobiology, University of Guelph, Guelph, Ontario N1G 2W1 Canada

^c^ Department of Biology, University of Padova, Padova, Italy

^d^ Department of Environmental Engineering, Technical University of Denmark, Miljoevej, Building 115, DK-2800 Kgs. Lyngby, Denmark

^e^ Department of General Biology, Federal University of Viçosa, Av. Peter Henry Rolfs, s/n, Campus Universitário, 36570-900, Viçosa, Minas Gerais, Brazil

^f^ Embrapa Maize and Sorghum, Rodovia MG 424, Sete Lagoas, Minas Gerais, Brazil

^g^ Department of Plant Patology, Federal University of Viçosa, Av. Peter Henry Rolfs, s/n, Campus Universitário, 36570-900, Viçosa, Minas Gerais, Brazil

^h^ Núcleo de Análise de Biomoléculas (NuBioMol), Center of Biological Sciences, Federal University of Viçosa, Viçosa, Minas Gerais, Brazi

**Supplementary table-1. vB_EcoM-UFV13 functional genomic annotation. Asterisks mean the best hit for a gene/protein found when no matches were identified on the reference Enterobacteria phage T4 (accession number NC_000866).**

|  | | | | | | | | |
| --- | --- | --- | --- | --- | --- | --- | --- | --- |
| **ORF** | **Predicted Protein & organism*** | **Genomic Coordinates** | **Strand** | **Protein mass**  **(kDa)** | **Protein pI** | **Gene & organism*** | **E-value** | **Identity**  **(%)** |
| 1 | RIIA | 0..2177 | Minus | 82.7 | 5.65 | *rIIA* | 0.0 | 98.6 |
| 2 | RIIA.1 | 2188..2391 | Minus | 8.1 | 6.58 | *rIIA.1* | 6.4e-47 | 98.5 |
| 3 | 39 | 2446..4263 | Minus | 68.3 | 7.20 | *39* | 0.0 | 98.5 |
| 4 | 39.1 | 4333..4593 | Minus | 9.2 | 7.71 | *39.1* | 9.4e-55 | 97.7 |
| 5 | Gp20 | 4599..4970 | Minus | 14.1 | 5.50 | *20*  (Shigella phage pSs-1) | 5.6e-75 | 95.1 |
| 6 | 39.2 | 4973..5149 | Minus | 6.7 | 8.39 | *39.2* | 3.8e-34 | 81.0 |
| 7 | ComC-α | 5152..5571 | Minus | 16.4 | 4.73 | *goF* = *comC-α* | 5.3e-79 | 83.7 |
| 8 | Cef | 5571..5786 | Minus | 8.4 | 5.0 | *cef* = *motC* | 2.4e-51 | 97.2 |
| 9 | Hypothetical Protein  (Escherichia coli O157 typing phage 7) | 5800..6069 | Minus | 10.2 | 5.76 | - | 5.6e-57 | 100.0 |
| 10 | MotB | 6167..6655 | Minus | 18.1 | 9.30 | *motB* | 9.1e-111 | 99.4 |
| 11 | MotB.1 | 6732..7265 | Minus | 20.0 | 4.92 | *motB.1* | 4e-22 | 63.3 |
| 12 | MotB.2 | 7268..7768 | Minus | 19.9 | 5.74 | *motB.2* | 2e-109 | 90.9 |
| 13 | DexA | 7832..8515 | Minus | 26.0 | 4.88 | dexA | 2.9e-164 | 98.7 |
| 14 | DexA.1 | 8515..8757 | Minus | 9.4 | 5.10 | *dexA.1* | 5e-55 | 91.3 |
| 15 | DexA.2 | 8750..8995 | Minus | 9.4 | 4.47 | *dexA.2* | 5.8e-57 | 97.5 |
| 16 | Hypothetical Protein | 8982..9242 | Minus | 9.9 | 8.79 | *30*  (Shigella phage pSs-1) | 1.1e-48 | 94.2 |
| 17 | Dda | 9249..10214 | Minus | 37.0 | 6.97 | *dda* = *sud* | 0.0 | 99.5 |
| 18 | Frameshift | 10298..10567 |  |  |  | Frameshift |  |  |
| 19 | Dda.1 | 10564..10875 | Minus | 12.0 | 9.47 | *dda.1* | 1e-67 | 98.1 |
| 20 | Srd | 10877..11623 | Minus | 29.0 | 9.99 | *srd* = *dda.2* | 1.3e-171 | 99.2 |
| 21 | RNA polymerase | 11746..12348 | Minus | 23.4 | 5.86 | *modB* | 6.5e-140 | 98.5 |
| 22 | ADP-ribosylase | 12345..12968 | Minus | 24.2 | 5.43 | *modA* | 5.4e-140 | 95.7 |
| 23 | ModA.2 | 13036..13218 | Minus | 7.0 | 4.27 | *modA.2* | 1.2e-44 | 98.3 |
| 24 | ModA.3 | 13227..13697 | Minus | 18.3 | 6.11 | *modA.3* | 4.3e-101 | 94.9 |
| 25 | ModA.4 | 13690..13842 | Minus | 5.6 | 5.73 | *modA.4* | 8.2e-33 | 98.0 |
| 26 | Srh | 13851..14054 | Minus | 8.0 | 6.27 | *srh* | 1.4e-49 | 98.5 |
| 27 | Mrh | 14029..14514 | Minus | 18.2 | 4.58 | *mrh* | 2.4e-83 | 77.0 |
| 28 | Mrh.1 | 14523..14864 | Minus | 12.6 | 3.85 | *mrh.1* | 9.4e-81 | 99.1 |
| 29 | Mrh.2 | 14864..15070 | Minus | 8.2 | 5.58 | *mrh.2* | 1.6e-49 | 97.1 |
| 30 | Soc | 15166..15411 | Minus | 9.3 | 5.62 | *soc* | 1.3e-60 | 97.5 |
| 31 | Soc.1 | 15428..15637 | Minus | 7.9 | 9.16 | *soc.1*  (Shigella phage Shfl2) | 8.8e-54 | 100.0 |
| 32 | Soc.2 | 15634..15834 | Minus | 7.8 | 9.94 | *soc.2*  (Shigella phage Shfl2) | 110e-48 | 100.0 |
| 33 | DNA Replication Protein | 15834..16352 | Minus | 20.2 | 5.16 | *56* | 3.4e-76 | 69.2 |
| 34 | Dam.1 | 16424..16624 | Plus | 7.1 | 10.00 | *dam.1* | 5.4e-44 | 98.5 |
| 35 | DNA Primase | 16621..17649 | Minus | 39.7 | 9.31 | *61* = *58* | 0.0 | 100.0 |
| 36 | Gp61.1 | 17652..17816 | Minus | 5.9 | 5.39 | *61.1* | 1.2e-36 | 96.3 |
| 37 | Gp61.2 | 17818..18174 | Minus | 13.8 | 4.83 | *61.2* | 2.1e-2 | 35.0 |
| 38 | Spackle periplasmic protein | 18187..18480 | Minus | 10.9 | 4.62 | *sp* | 5.4e-62 | 97.9 |
| 39 | Hypothetical Protein (Enterobacteria phage ime09) | 18584..18766 | Minus | 7.1 | 4.84 | - | 510e-45 | 98.3% |
| 40 | Hypothetical Protein  (Shigella phage Shfl2) | 18831..19085 | Minus | 9.6 | 5.81 | - | 17e-63 | 100.0 |
| 41 | Gp61.4 | 19149..19391 | Minus | 9.5 | 10.29 | *61.4* | 4.5e-55 | 96.1 |
| 42 | Dmd | 19393..19575 | Minus | 7.0 | 5.24 | *dmd* | 3.8e-43 | 100.0 |
| 43 | DNA Helicase | 19634..21061 | Minus | 53.5 | 5.44 | *41* | 0.0 | 99.8 |
| 44 | Head Formation Protein | 21071..21415 | Minus | 13.2 | 4.99 | *40* | 1.0e-72 | 99.1 |
| 45 | UvsX | 21408..22589 | Minus | 44.0 | 5.35 | *uvsX* = *fdsA* | 0.0 | 90.3 |
| 46 | Beta-glucosyl-HMC-alpha-glucosyl-transferase | 22667..23509 | Minus | 32.3 | 7.65 | *β-gt* (Enterobacterio phage RB27) | 0.0 | 98.9 |
| 47 | dCMP hydroxymethylase | 23506..24246 | Minus | 28.5 | 5.43 | *42* | 2.1e176 | 97.1 |
| 48 | Inner Membrane Protein | 24400..24651 | Minus | 93.0 | 9.40 | *imm* | 9.9e-87 | 98.8 |
| 49 | Imm Intergenic Region | 24659..25039 | Minus | 14.2 | 7.55 | *imm.1* | 280e-90 | 99.2 |
| 50 | Hypothetical Protein  (Enterobacteria phage T2) | 25052..25303 | Minus | 9.3 | 9.60 | - | 10e-60 | 98.8 |
| 51 | DNA polymerase | 25479..28175 | Minus | 103.5 | 5.96 | *43* | 0.0 | 99.6 |
| 52 | Hypothetical Protein  (Enterobacteria phage RB18) | 28254..28475 | Minus | 8.4 | 6.25 | - | 9.7e-53 | 98.6 |
| 53 | RegA | 28477..28845 | Minus | 14.6 | 8.97 | *regA* | 1.2e-83 | 100.0 |
| 54 | Clamp loader small subunit | 28847..29410 | Minus | 21.3 | 7.67 | *62* | 2.1e-129 | 99.5 |
| 55 | Clamp loader small subunit | 29412..30371 | Minus | 35.7 | 6.72 | *44* | 0.0 | 100.0 |
| 56 | DNA polymerase clamp | 30423..31109 | Minus | 24.8 | 4.89 | *45* | 3.6e-156 | 99.1 |
| 57 | RNA polymerase-binding protein | 31165..31554 | Minus | 14.7 | 6.83 | *rpbA* | 16e-87 | 100.0 |
| 58 | Hypothetical Protein | 31564..31752 | Minus | 7.5 | 5.55 | *45.2* | 7.3e-45 | 98.4 |
| 59 | Recombination-related endonuclease | 31808..33448 | Minus | 63.6 | 7.13 | *46* | 0.0 | 95.2 |
| 60 | Hypothetical Protein | 33487..33693 | Minus | 8.1 | 4.24 | *46.1* | 5.1e-50 | 98.5 |
| 61 | Hypothetical Protein | 33674..33937 | Minus | 10.2 | 4.21 | 46.2 | 1.2e-58 | 97.7 |
| 62 | Recombination-related endonuclease | 33934..34953 | Minus | 39.1 | 4.94 | 47 | 0.0 | 96.2 |
| 63 | Alpha glucosyl transferase | 35130..36332 | Minus | 46.8 | 6.05 | *α-gt* | 0.0 | 91.5 |
| 64 | Alpha glucosyl transferase.2 | 36399..36569 | Minus | 6.4 | 9.3 | *α-gt.2* | 1.7e-28 | 88.7 |
| 65 | Alpha glucosyl transferase.3 | 36573..36776 | Minus | 7.9 | 9.42 | *α-gt.3* | 9.7e-50 | 98.5 |
| 66 | Alpha glucosyl transferase.4 | 36745..37062 | Minus | 12.4 | 8.78 | *α-gt.4* | 1.3e-66 | 100.0 |
| 67 | Alpha glucosyl transferase.5 | 37064..37252 | Minus | 7.4 | 4.10 | *α-gt.5* | 1.8e-44 | 98.5 |
| 68 | RNA polymerase sigma fator for late transcription | 37266..37823 | Minus | 21.5 | 5.44 | *55* | 9.6e-132 | 100.0 |
| 69 | Hypothetical Protein  (Shigella phage Shfl2) | 37902..38171 | Minus | 10.7 | 5.64 | - | 8.2e-60 | 100.0 |
| 70 | MobC | 38168..38383 | Minus | 8.0 | 3.69 | *55.1* | 1.6e-49 | 97.1 |
| 71 | Gp55.2 | 38386..38712 | Minus | 12.7 | 9.70 | *55.2* | 3.6e-68 | 99.1 |
| 72 | Gp55.3 | 38765..38965 | Minus | 7.7 | 7.89 | *55.3* | 3.1e-35 | 74.2 |
| 73 | Gp55.4 | 38966..39097 | Minus | 5.1 | 9.60 | *55.4* | 3.8e-34 | 97.7 |
| 74 | Gp55.5 | 39105..39398 | Minus | 11.8 | 9.79 | *55.5* | 2.8e-64 | 100.0 |
| 75 | Gp55.6 | 39391..39573 | Minus | 6.9 | 9.37 | *55.6* | 2.4e-38 | 93.3 |
| 76 | Glutaredoxin | 39732..40040 | Minus | 11.7 | 9.14 | *nrdH* | 2.7e-67 | 100.0 |
| 77 | Gp55.8 | 40043..40255 | Minus | 7.9 | 9.22 | *55.8* | 2.9e-48 | 100.0 |
| 78 | Gp55.9 | 40265..40378 | Minus | 4.4 | 4.54 | *nrdG* = *55.9* | 8.8e-30 | 100.0 |
| 79 | Ribonucleotide reductase of class III (anaerobic), activating protein | 40371..40841 | Minus | 18.1 | 5.58 | *nrdG* | 9.6e-93 | 80.1 |
| 80 | Ribonucleotide reductase of class III (anaerobic), large subunit | 40838..42655 | Minus | 67.9 | 6.59 | *nrdD* = *sunY* | 0.0 | 99.5 |
| 81 | Gp49 | 42652..43125 | Minus | 18.1 | 8.80 | *49* | 7.7e-112 | 100.0 |
| 82 | Pin | 43167..43652 | Minus | 18.8 | 4.48 | *pin* | 3.4e-110 | 96.3 |
| 83 | Gp49.1 | 43636..43791 | Minus | 6.1 | 3.94 | *49.1* | 4e-40 | 100.0 |
| 84 | Gp49.2 | 43776..44096 | Minus | 12.5 | 4.48 | *49.2* | 2.9e-71 | 97.2 |
| 85 | Hypothetical Protein | 44107..44277 | Minus | 6.5 | 4.07 | *95*  (Shigella phage pSs-1) | 130e-36 | 91.1 |
| 86 | Hypothetical Protein | 44280..44495 | Minus | 8.1 | 7.79 | *96*  (Shigella phage pSs-1) | 3.1e-51 | 98.6 |
| 87 | Thioredoxin | 44492..44755 | Minus | 10.0 | 6.71 | *nrdC* | 6.6e-58 | 100.0 |
| 88 | NrdC.1 | 44757..44999 | Minus | 9.4 | 7.91 | *nrdc.1* | 1.7e-59 | 100.0 |
| 89 | NrdC.2 | 44986..45303 | Minus | 12.2 | 6.07 | *nrdc.2* | 2.8e-63 | 90.5 |
| 90 | NrdC.3 | 45300..46229 | Minus | 36.0 | 9.10 | *nrdc.3* | 3.6e-124 | 60.6 |
| 91 | NrdC.4 | 46282..47313 | Minus | 40.0 | 6.00 | *nrdC.4* | 0.0 | 96.4 |
| 92 | NrdC.5 | 47341..48366 | Minus | 39.9 | 9.32 | *nrdC.5* | 0.0 | 94.2 |
| 93 | NrdC.6 | 48375..49262 | Minus | 33.7 | 9.15 | *nrdC.6* | 0.0 | 96.6 |
| 94 | NrdC.7 | 49270..49677 | Minus | 15.4 | 5.28 | *nrdC.7* | 7.5e-81 | 88.7 |
| 95 | NrdC.8 | 49733..50260 | Minus | 20.7 | 6.65 | *nrdC.8* | 5.3e-119 | 98.9 |
| 96 | NrdC.9 | 50321..50623 | Minus | 11.9 | 9.68 | *nrdC.9* | 2.3e-67 | 99.0 |
| 97 | NrdC.10 | 50725..51693 | Minus | 36.4 | 5.01 | *nrdC.10* | 0.0 | 99.7 |
| 98 | NrdC.11 | 51809..52819 | Minus | 38.9 | 6.39 | *nrdC.11* | 0.0 | 99.4 |
| 99 | Hypothetical protein  (Enterobacteria phage ime09) | 52819..53280 | Minus | 17.9 | 9.33 | - | 420e-111 | 99.3 |
| 100 | MobD.1 | 53283..53804 | Minus | 19.2 | 4.99 | *mobD.1* | 1.1e-1 | 25.6 |
| 101 | MobD.1 | 53811..54368 | Minus | 21.7 | 5.68 | *mobD.1* | 1.2e-109 | 87.5 |
| 102 | MobD.2a | 54536..54709 | Minus | 6.7 | 5.27 | *mobD.2a* | 5.2e-37 | 96.5 |
| 103 | MobD.3 | 54699..54893 | Minus | 7.6 | 5.04 | *mobD.3* | 4.3e-44 | 95.3 |
| 104 | MobD.4 | 54896..55099 | Minus | 7.4 | 4.29 | *mobD.4* | 6.4e-29 | 77.6 |
| 105 | MobD.5 | 55099..55287 | Minus | 7.1 | 4.19 | *mobD.5* | 9.7e-43 | 100.0 |
| 106 | RI.-1 | 55383..55769 | Minus | 14.6 | 5.53 | *rI.-1* | 1.2e-86 | 100.0 |
| 107 | RI | 55766..56059 | Minus | 11.1 | 4.90 | *rI* | 1.5e-61 | 99.0 |
| 108 | RI.1 | 56072..56284 | Minus | 8.2 | 10.19 | *rI.1* | 4.4e-49 | 100.0 |
| 109 | Thymidine kinase | 56327..56908 | Minus | 21.6 | 6.22 | *tk* | 5.6e-135 | 100.0 |
| 110 | Thymidine kinase.2 | 56918..57103 | Minus | 7.3 | 4.41 | *tk.2* | 5.4e-17 | 54.1 |
| 111 | Hypothetical protein  (Enterobacteria phage RB5) | 57100..57273 | Minus | 6.6 | 5.68 | - | 9.3e-38 | 100.0 |
| 112 | Hypothetical protein  (Escherichia phage ECML-134) | 57270..57476 | Minus | 7.8 | 6.68 | - | 1.1e-57 | 100.0 |
| 113 | Thymidine kinase.3 | 57473..57685 | Minus | 8.5 | 8.83 | *tk.3* | 2.1e-53 | 92.9 |
| 114 | Thymidine kinase.4 | 57657..58136 | Minus | 17.5 | 5.27 | *tk.4* | 2.6e-60 | 57.4 |
| 115 | Valyl-tRNA synthetase modifier | 58133..58480 | Minus | 13.0 | 8.95 | *vs* | 9.5e-77 | 100.0 |
| 116 | Endoribonuclease RegB | 58473..59018 | Minus | 20.6 | 9.76 | *regB* | 4.5e-128 | 100.0 |
| 117 | Site-specific RNA endonuclease | 59026..59487 | Minus | 17.9 | 8.93 | *regB* | 2.8e-105 | 99.3 |
| 118 | Vs.3 | 59547..59825 | Minus | 10.9 | 5.45 | *vs.3* | 4.7e-58 | 100.0 |
| 119 | Vs.4 | 59825..60091 | Minus | 10.2 | 4.75 | *vs.4* | 8.2e-57 | 97.7 |
| 120 | Vs.5 | 60084..60305 | Minus | 8.2 | 4.14 | *vs.5* | 2.1e-55 | 97.3 |
| 121 | Vs.6 | 60305..60667 | Minus | 13.8 | 5.80 | *vs.6* | 1.3e-79 | 100.0 |
| 122 | Vs.7 | 60675..61004 | Minus | 12.8 | 8.89 | *vs.7* | 2.7e-73 | 98.2 |
| 123 | Vs.8 | 61001..61570 | Minus | 21.5 | 8.82 | *vs.8* | 2.6e-123 | 95.1 |
| 124 | Internal Head Protein 3 | 61707..62282 | Minus | 21.4 | 9.56 | *ip3* | 9e-124 | 91.7 |
| 125 | Internal Head Protein 10 | 62297..62611 | Minus | 12.0 | 9.49 | *ip10*  (Enterobacteria phage Pol) | 1.8e-60 | 97.9 |
| 126 | Hypothetical protein  (Escherichia phage Av-05) | 62675..62827 | Minus | 5.3 | 4.60 | - | 6.2e-28 | 93.8 |
| 127 | Hypothetical protein  (Shigella phage Shf125875) | 62799..62927 | Minus | 5.0 | 9.22 | - | 6.3e-24 | 94.9 |
| 128 | Endonuclease V | 62940..63353 | Minus | 16.0 | 9.49 | *denV* | 3.1e-84 | 91.2 |
| 129 | Internal Head Protein 5 | 63412..63693 | Minus | 10.4 | 5.54 | *ipV* | 3.4e-17 | 51.2 |
| 130 | Lysozyme | 63690..64178 | Minus | 18.3 | 9.66 | *e* | 7e-106 | 95.0 |
| 131 | Nudix Hydrolase.1 | 64216..64656 | Minus | 17.0 | 5.18 | *nudE* = *e.1* | 1.7e-101 | 97.9 |
| 132 | Nudix Hydrolase.2 | 64653..65141 | Minus | 19.0 | 8.26 | *e.2* | 2.8e-53 | 84.2 |
| 133 | Nudix Hydrolase.3 | 65138..65500 | Minus | 14.1 | 8.83 | *e.3* | 2.7e-61 | 80.0 |
| 134 | Nudix Hydrolase.4 | 65482..65874 | Minus | 15.2 | 9.67 | *e.4* | 6.5e-80 | 90 |
| 135 | Nudix Hydrolase.5 | 65843..66445 | Minus | 23.6 | 5.10 | *e.5* | 1.2e-133 | 93.5 |
| 136 | Nudix Hydrolase.6 | 66493..67086 | Minus | 22.0 | 6.06 | *e.6* | 3.5e-126 | 98.5 |
| 137 | Nudix Hydrolase.7 | 67144..67479 | Minus | 12.9 | 4.25 | *e.7* | 14e-69 | 91.0 |
| 138 | Hypothetical protein  (Shigella phage SH7) | 67524..67688 | Minus | 6.1 | 3.96 | - | 4.5e-39 | 98.1 |
| 139 | Nudix Hydrolase.8 | 67758..68021 | Minus | 10.1 | 4.46 | *e.8* | 3.3e-58 | 98.9 |
| 140 | Hypothetical protein  (Shigella phage Shfl2) | 68260..68826 | Minus | 21.0 | 9.43 | - | 2.3e-126 | 99.5 |
| 141 | Hypothetical protein  (Enterobacteria phage RB32) | 68945..69418 | Minus | 17.2 | 9.46 | - | 1.9e-102 | 98.7 |
| 142 | tRNA.2 | 70737..71024 | Minus | 11.2 | 4.93 | *tRNA.2* | 1.9e-61 | 96.8 |
| 143 | tRNA.3 | 71027..71431 | Minus | 15.8 | 4.67 | *tRNA.3* | 2.8e-86 | 85.3 |
| 144 | tRNA.4 | 71432..71617 | Minus | 6.5 | 7.80 | *tRNA.4* | 1.9e-40 | 100.0 |
| 145 | Internal Protein I | 71694..71981 | Minus | 10.1 | 8.93 | *ipI* | 2e-58 | 98.9 |
| 146 | Gp57B | 72054..72509 | Minus | 17.1 | 5.14 | *57B* | 1.7e-105 | 100.0 |
| 147 | Chaperone for tail fiber formation | 72509..72763 | Minus | 9.1 | 4.31 | *57A* | 2e-52 | 94.0 |
| 148 | Deoxynucleotide monophosphate kinase | 72763..73476 | Minus | 26.8 | 5.14 | *1* | 8.8e-165 | 96.7 |
| 149 | Tail completion and sheath stabilizer protein | 73526..74056 | Minus | 19.7 | 4.39 | *3* | 7.8e-124 | 100.0 |
| 150 | DNA end protector during packaging | 74162..74986 | Minus | 31.6 | 10.12 | *2* = *64* | 0.0 | 100.0 |
| 151 | Head completion protein | 74986..75438 | Minus | 17.6 | 9.78 | *4* = *50* = *65* | 3.5e-109 | 100.0 |
| 152 | Baseplate wedge subunit | 75486..76076 | Plus | 22.9 | 5.92 | *53* | 7.6e-144 | 100.0 |
| 153 | Base plate lysozyme; hub component | 76060..77787 | Plus | 63.1 | 5.35 | *5* | 0.0 | 99.3 |
| 154 | 5.1 | 77780..78316 | Plus | 20.0 | 4.45 | *5.1* | 7.5e-110 | 96.3% |
| 155 | 5.4 | 78317..78610 | Plus | 10.2 | 8.60 | *5.4* | 8.2e-64 | 100.0 |
| 156 | Baseplate wedge component | 78619..80601 | Plus | 74.4 | 4.62 | *6* | 0.0 | 99.5 |
| 157 | Baseplate wedge component | 80658..83696 | Plus | 116.9 | 4.98 | *7* | 0.0 | 98.5 |
| 158 | Baseplate wedge component | 83689..84693 | Plus | 37.9 | 4.58 | *8* | 0.0 | 99.1 |
| 159 | Base plate wedge component, tail fiber  socket, trigger for tail sheath  contraction | 84757..85623 | Plus | 30.9 | 5.01 | *9* | 0.0 | 99.3 |
| 160 | Baseplate wedge subunit and tail pin (T4-like gp10) | 85623..87431 | Plus | 66.3 | 4.41 | *10* | 0.0 | 99.8 |
| 161 | Baseplate wedge subunit and tail pin (T4-like gp11) | 87431..88090 | Plus | 23.6 | 5.30 | *11* | 3.8e-151 | 98.2 |
| 162 | Short tail fibers | 88087..89670 | Plus | 55.9 | 7.02 | *12* | 0.0 | 96.6 |
| 163 | Whiskers, facilitate long tail fiber | 89667..91130 | Plus | 51.8 | 4.54 | *wack* | 0.0 | 97.3 |
| 164 | Head completition, neck hetero-dimeric protein (T4-like gp13) | 91163..92092 | Plus | 34.7 | 5.02 | 13 | 0.0 | 99.7 |
| 165 | Head completition, neck hetero-dimeric protein | 92094..92864 | Plus | 29.5 | 4.57 | 14 | 0.0 | 99.6 |
| 166 | Tail sheath stabilization protein | 92906..93724 | Plus | 31.5 | 4.88 | *15* | 0.0 | 100.0 |
| 167 | Terminase, small subunit | 93733..94227 | Plus | 18.3 | 4.55 | *16* | 4.5e-112 | 100.0 |
| 168 | Terminase, large subunit | 94211..96043 | Plus | 69.6 | 5.58 | *17* | 0.0 | 99.7 |
| 169 | Tail sheath stabilization monomer | 96075..98054 | Plus | 71.3 | 4.82 | *18* | 0.0 | 98.8 |
| 170 | Tail fibers | 98171..98662 | Plus | 18.4 | 4.67 | *19* | 1.4e-115 | 100.0 |
| 171 | Portal vertex of the head | 98746..100320 | Plus | 61.0 | 5.36 | *20* | 0.0 | 99.6 |
| 172 | Prohead core protein | 100320..100559 | Plus | 8.9 | 3.96 | *67* | 4.7e-55 | 96.3 |
| 173 | Capsid and scaffold | 100559..100984 | Plus | 15.8 | 10.10 | *68* | 4.9e-90 | 99.3 |
| 174 | Prohead assembly (scaffolding) protein | 100984..101622 | Plus | 23.2 | 4.98 | *21* | 3.9e-152 | 99.5 |
| 175 | Prohead assembly (scaffolding) protein | 101653..102462 | Plus | 29.8 | 4.56 | *22* | 4.2e-176 | 98.1 |
| 176 | Major capsid protein | 102481..104046 | Plus | 56.0 | 5.34 | *23* | 0.0 | 98.8 |
| 177 | Capsid vertex | 104130..105413 | Plus | 47.0 | 4.70 | *os = 24* | 0.0 | 99.3 |
| 178 | Second RNA ligase | 105443..106447 | Minus | 37.6 | 5.65 | *rnlB = 24.1* | 0.0 | 99.1 |
| 179 | Gp24.2 | 106457..106735 | Minus | 10.9 | 4.87 | *24.2* | 5.8e-59 | 96.7 |
| 180 | Gp24.3 | 106722..106925 | Minus | 7.8 | 10.34 | *24.3* | 1.9e-33 | 83.6 |
| 181 | Capsid and scaffold | 107027..108157 | Minus | 40.7 | 4.51 | *hoc* = *eph* | 0.0 | 88.0 |
| 182 | Inh inhibitor of gp21 prohead protease | 108167..108847 | Minus | 25.5 | 4.43 | *inh* = *lip* | 9.3e-158 | 99.1 |
| 183 | DNA helicase | 108898..110409 | Plus | 57.9 | 9.29 | *uvsW* = *dar* | 0.0 | 98.8 |
| 184 | DNA helicase | 110435..110665 | Plus | 8.8 | 4.24 | *uvsW* = *dar* | 9.5e-50 | 100.0 |
| 185 | UvsY intergenic region | 110721..110888 | Minus | 6.0 | 4.44 | *uvsY.-2* | 2.9e-39 | 100.0 |
| 186 | UvsY.-1 | 110917..111141 | Minus | 8.9 | 5.02 | *uvsY.-1* | 2.4e-56 | 98.6 |
| 187 | Single Stranded DNA-binding protein | 111141..111554 | Minus | 15.7 | 7.75 | *uvsY* | 1.8e-90 | 98.5 |
| 188 | Baseplate wedge subunit | 111621..112019 | Minus | 15.1 | 4.57 | *25* | 7.9e-87 | 98.5 |
| 189 | Baseplate hub assembly chaperone | 112019..112645 | Minus | 23.8 | 5.56 | *26* | 1.1e-149 | 99.5 |
| 190 | Baseplate | 112696..113445 | Plus | 29.2 | 6.02 | *51* | 7.2e-174 | 96.0 |
| 191 | Baseplate hub subunit | 113445..114620 | Plus | 44.3 | 5.20 | *27* | 0.0 | 99.2 |
| 192 | Baseplate hub | 114640..115098 | Plus | 17.3 | 5.01 | *28* | 9e-107 | 99.3 |
| 193 | Baseplate hub | 115095..116867 | Plus | 64.2 | 5.05 | *29* | 0.0 | 95.8 |
| 194 | Baseplate tube cap (T4-like gp48) | 116876..117970 | Plus | 39.6 | 8.71 | *48* | 0.0 | 97.8 |
| 195 | Tail assembly | 117970..118935 | Plus | 34.8 | 5.08 | *54* | 0.0 | 97.8 |
| 196 | Alt.-3 | 118964..119254 | Minus | 10.7 | 4.67 | *alt.-3* | 1.9e-59 | 100.0 |
| 197 | Alt.-2 | 119315..121372 | Minus | 76.0 | 6.01 | *alt.-2* | 2e-147 | 41.5 |
| 198 | Alt | 121376..123469 | Minus | 77.8 | 5.97 | *alt* | 0.0 | 70 |
| 199 | Alt.1 | 123522..123710 | Minus | 7.1 | 4.50 | *alt.1* | 4.7e-41 | 90.3 |
| 200 | DNA ligase | 123707..125170 | Minus | 55.2 | 6.14 | *30* = *lig* | 0.0 | 99.6 |
| 201 | Gp30.1 | 125167..125436 | Minus | 10.8 | 7.82 | 30.1 | 7.1e-61 | 98.9 |
| 202 | Gp30.2 | 125436..126275 | Minus | 32.4 | 5.93 | 30.2 | 0.0 | 96.8 |
| 203 | Gp30.3 | 126272..126652 | Minus | 14.0 | 9.33 | 30.3 | 2e-80 | 96.8 |
| 204 | Gp30.4 | 126723..126938 | Minus | 8.2 | 4.94 | 30.4 | 1.7e-16 | 50.7 |
| 205 | Gp30.6 | 126943..127230 | Minus | 10.8 | 6.26 | *30.6* | 1.2e-59 | 96.8 |
| 206 | Gp30.7 | 127272..127637 | Minus | 14.1 | 6.05 | *30.7* | 1.9e-84 | 98.3 |
| 207 | Gp30.8 | 127705..128037 | Minus | 12.8 | 6.11 | *30.8* | 2.9e-73 | 97.3 |
| 208 | Gp30.9 | 128148..128366 | Minus | 8.1 | 11.33 | *30.9* | 2.1e-50 | 98.6 |
| 209 | RIII | 128445..128693 | Minus | 9.3 | 8.06 | *rIII* | 3.2e-61 | 100.0 |
| 210 | Gp31 | 128841..129176 | Minus | 12.0 | 5.30 | *31* | 1.3e-74 | 100.0 |
| 211 | Gp31.1 | 129233..129541 | Minus | 11.4 | 9.35 | *31.1* | 2.9e-64 | 100.0 |
| 212 | Gp31.2 | 129542..129778 | Minus | 9.3 | 9.78 | *31.2* | 1.3e-56 | 96.2 |
| 213 | tRNA-specific adenosine deaminase | 129778..130359 | Minus | 21.2 | 8.04 | *cd* | 2.3e-141 | 99.5 |
| 214 | Cd.1 | 130356..130694 | Minus | 12.8 | 7.87 | *cd.1* | 1.1e-71 | 99.1 |
| 215 | Cd.2 | 130691..130930 | Minus | 8.9 | 5.31 | *cd.2* | 1.1e-47 | 98.6 |
| 216 | Cd.3 | 130992..131267 | Minus | 10.1 | 4.90 | *cd.3* | 3.3e-55 | 98.9 |
| 217 | Cd.4 | 131270..131470 | Minus | 7.9 | 4.35 | *cd.4* | 1.2e-47 | 97 |
| 218 | Cd.5 | 131463..131660 | Minus | 7.5 | 6.71 | *cd.5* | 1.8e-47 | 97 |
| 219 | 3’-phosphatase, 5’-polynucleotide kinase | 131660..132568 | Minus | 34.8 | 8.62 | *pseT* | 0.0 | 95.0 |
| 220 | Hypothetical protein  (Escherichia phage slur14) | 132565..132885 | Minus | 12.1 | 8.55 |  | 1.8e-68 | 100.0 |
| 221 | PseT.1 | 132882..133112 | Minus | 8.9 | 8.02 | *pseT.1* | 1.4e-55 | 98.1 |
| 222 | PseT.2 | 133109..133408 | Minus | 11.6 | 8.76 | *pseT.2* | 6.2e-67 | 99.0 |
| 223 | PseT.3 | 133405..133758 | Minus | 13.0 | 8.89 | *pseT.3* | 9.7e-68 | 92.3 |
| 224 | Alc | 133749..134252 | Minus | 19.0 | 6.42 | *alc* | 9.9e-113 | 97.6 |
| 225 | RNA ligase I | 134317..135441 | Minus | 43.4 | 5.02 | *rnlA* = *63* | 0.0 | 98.4 |
| 226 | Endonuclease II | 135494..135904 | Minus | 15.7 | 9.21 | *denA* | 2.9e-91 | 99.3 |
| 227 | NrdB (beta subunit) | 135932..137101 | Minus | 45.2 | 5.13 | *nrdB* | 0.0 | 91.3 |
| 228 | NrdB (alfa subunit) | 137153..139417 | Minus | 86.0 | 5.87 | *nrdB* | 0.0 | 98.4 |
| 229 | NrdA.1 | 139408..139734 | Minus | 12.4 | 9.22 | *nrdA.1* | 7.2e-73 | 96.3 |
| 230 | NrdA.2 | 139688..139951 | Minus | 9.9 | 5.28 | *nrdA.2* | 1e-54 | 96.6 |
| 231 | Thymidylate synthase | 139975..140835 | Minus | 32.9 | 8.57 | *td* | 0.0 | 94.1 |
| 232 | Thymidylate synthse.1 | 140881..141228 | Minus | 13.7 | 7.69 | *td.1*  (Enterobacteria phage T6) | 42e-81 | 98.3 |
| 233 | frd | 141248..141829 | Minus | 21.7 | 5.58 | *frd* | 6.1e-132 | 97.4 |
| 234 | Hypothetical protein  (Enterobacteria phage RB9) | 141829..142074 | Minus | 9.6 | 4.05 | - | 870e-66 | 98.8% |
| 235 | Frd.1 | 142085..142327 | Minus | 9.4 | 4.91 | *frd.1* | 3.7e-62 | 97.5 |
| 236 | Frd.2 | 142382..142747 | Minus | 14.4 | 5.41 | *frd.2* | 3.6e-48 | 65.1 |
| 237 | Frd.3 | 142792..143019 | Minus | 8.6 | 3.86 | *frd.3* | 4.2e-37 | 70.7 |
| 238 | Single-stranded DNA-binding protein | 143164..144072 | Minus | 33.5 | 4.87 | *32* | 0.0 | 97.7 |
| 239 | DNA Helicase loader | 144172..144825 | Minus | 26.0 | 9.33 | *59* | 2.8e-152 | 99.5 |
| 240 | Gp33 | 144822..145160 | Minus | 12.8 | 4.47 | *33* | 1e-69 | 98.2 |
| 241 | Double-stranded DNA-binding protein | 145138..145407 | Minus | 10.3 | 5.04 | *dsbA* | 1.4e-55 | 100.0 |
| 242 | Ribonuclease H | 145416..146333 | Minus | 35.5 | 8.61 | *rnh* = *das* | 0.0 | 99.7 |
| 243 | Long Tail fiber proximal subunit | 146438..150307 | Plus | 140.0 | 5.49 | *34* | 0.0 | 95.6 |
| 244 | Hinge connector of long tail fiber proximal connector | 150316..151431 | Plus | 40.2 | 5.27 | *35* | 0.0 | 97.6 |
| 245 | Hinge connector of long tail fiber distal connector | 151494..152156 | Plus | 23.2 | 6.82 | *36* | 5.9e-132 | 85.5 |
| 246 | Tail fibers | 152165..156025 | Plus | 137.8 | 8.30 | *37* | 0.0 | 55.2 |
| 247 | Assembly catalyst of distal tail fiber | 156056..156847 | Plus | 25.5 | 9.79 | *38*  (Shigella phage Shfl2) | 0.0 | 99.2 |
| 248 | Holin | 156879..157535 | Plus | 25.1 | 7.70 | *t* = *rV* = *stII* | 9.2e-154 | 99.1 |
| 249 | Anti-sigma 70 protein | 157536..157808 | Minus | 10.6 | 5.42 | *asiA* | 1.1e-55 | 98.9 |
| 250 | asiA.1 | 157821..157973 | Minus | 5.8 | 4.92 | *asiA.1* | 2e-30 | 94.0 |
| 251 | Inhibitor of MrcBC | 157970..158248 | Minus | 10.8 | 4.48 | *arn* | 3.3e-58 | 97.8 |
| 252 | Hypothetical protein  (Shigella phage SHBML-50-1) | 158238..158357 | Minus | 4.6 | 10.20 | - | 8.3e-28 | 100 |
| 253 | Arn.1 | 158332..158463 | Minus | 5.2 | 8.57 | *arn.1* | 3.8e-34 | 97.7 |
| 254 | Arn.2 | 158534..158830 | Minus | 11.3 | 8.93 | *arn.2* | 2.4e-63 | 96.9 |
| 255 | Arn.3 | 158830..159291 | Minus | 17.9 | 5.31 | *arn.3* | 1.5e-104 | 98.0 |
| 256 | Arn.4 | 159288..159617 | Minus | 12.7 | 9.24 | *arn.4* | 4.7e-74 | 99.1 |
| 257 | MotA | 159628..160263 | Minus | 23.5 | 7.78 | *motA* = *sip* | 1.2e-138 | 97.6 |
| 258 | Uncharacterized 4.8 kDa protein in motA-Gp52 intergenic region | 160390..160539 | ααMinus | 4.8 | 9.50 | *motA.1* | 9.5e-37 | 100.0 |
| 259 | DNA Topoisomerase | 160536..161864 | Minus | 50.4 | 8.45 | *52* | 0.0 | 99.3 |
| 260 | Ac | 162002..162160 | Minus | 5.5 | 4.21 | *ac* | 3.7e-32 | 96.1 |
| 261 | Nucleoid disruption protein | 162248..162706 | Minus | 17.1 | 10.05 | *ndd* = D2b | 5.1e-100 | 96.7 |
| 262 | Ndd.1 | 162767..162982 | Minus | 8.1 | 4.22 | *ndd.1* | 3.4e-49 | 95.8 |
| 263 | Ndd.2 | 162991..163116 | Minus | 5.0 | 5.75 | *ndd.2* | 1.3e-30 | 97.2 |
| 264 | Ndd.3 | 163098..163295 | Minus | 7.4 | 9.40 | *ndd.3* | 3.6e-21 | 95.0 |
| 265 | Ndd.5 | 163481..163612 | Minus | 5.0 | 4.65 | *ndd.5* | 1.6e-22 | 96.9 |
| 266 | Hypothetical protein  (Enterobacteria phage RB9) | 163691..163996 | Minus | 11.6 | 8.69 | - | 84e-39 | 100.0 |
| 267 | Endonuclease IV | 163978..164535 | Minus | 21.1 | 6.89 | *denB* | 2e-133 | 98.4 |
| 268 | denB.1 | 164598..164792 | Minus | 7.5 | 6.21 | *denB.1* | 8.6e-40 | 93.8 |
| 269 | RIIB | 164821..165759 | Minus | 35.6 | 6.26 | rIIB | 0.0 | 98.7 |

Supplementary table-2. Functional categorization of vB_EcoM-UFV13 genes based on the reference Enterobacteria phage T4.

| **Functional category** | **ORFs** |
| --- | --- |
| **Transcription** | 7, 10, 11, 12, 19, 20, 22, 23, 24, 25, 26, 27, 28,29, 57, 58, 68, 70, 71, 72, 73, 74, 75, 87, 88, 89, 90, 91, 92, 93, 94, 95, 96, 97, 98, 240, 241, 249, 250, 257 |
| **Translation** | 8, 21, 42, 53, 115, 116, 117, 118, 119, 120, 121, 122, 123, 178 |
| **Nucleotide metabolism** | 33, 47, 76, 78, 79, 80, 87, 110, 113, 114, 131, 132, 133, 134, 135, 136, 137, 139, 148, 213, 214, 215, 216, 217, 218, 219, 221, 226, 227, 228, 229, 230, 231, 232, 233, 235, 236, 237, 267, 268 |
| **DNA replication, recombination, repair, packaging, and**  **processing** | 3, 4, 6, 13, 14, 15, 17, 35, 36, 37, 43, 45,51,54, 55, 56, 59, 60, 61, 62, 81, 83, 84, 128, 167, 168, 183, 184, 185, 186, 187, 200, 201, 202, 203, 204, 205, 206, 207, 208, 238, 239, 242, 259 |
| **Virion proteins** | 30, 31, 32, 124, 125, 129, 145, 149, 150, 151, 152, 153, 156, 157, 158, 159, 160, 161, 162, 163, 164, 165, 166, 169, 170, 171, 172, 173, 174, 175, 176, 177, 179, 180, 181, 182, 188, 189, 191, 192, 193, 194, 195, 225, 243, 244, 245, 246 |
| **Chaperonins/assembly catalysts** | 44, 147, 174, 175, 190, 209, 210, 211, 212, 225, 247 |
| **Host or phage interactions** | 1, 2, 34, 46, 48, 49, 63, 64, 65, 66, 67, 82, 219, 225, 251, 253, 254, 255, 256, 260, 269 |
| **Host or phage interactions** | 1, 2, 34, 46, 48, 49, 63, 64, 65, 66, 67, 82, 219, 225, 251, 253, 254, 255, 256, 260, 269 |
| **Host alteration/shutoff** | 127, 196, 197, 198, 199, 224, 261, 262, 263, 264, 265 |
| **Homing endonucleases and homologs** | 100, 101, 102, 103, 104, 105 |
| **Predicted integral membrane or periplasmic proteins** | 77, 144, 222, 223, 252 |
| **Unknown function** | 5, 9, 16, 39, 40, 41, 50, 52, 69, 85, 86, 99, 111, 112, 126, 138, 140, 141, 220, 234, 258, 266 |

**Supplementary figure-1.**
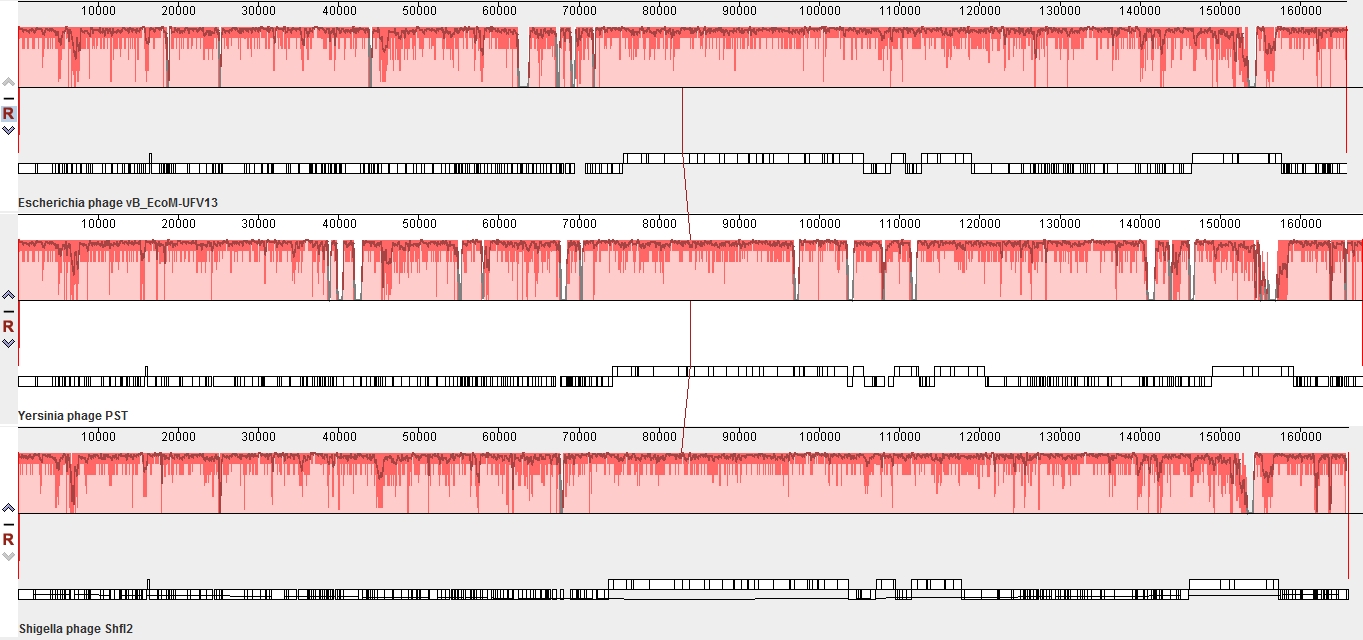
vB_EcoM-UFV13, *Yersinia* phage PST and *Shigella* phage Shfl2 genomes alignment with progressive Mauve showing collinear blocks between the genomes. In each alignment is possible observe base pairs scale and histogram with degree of similarity. BLASTn analyze revealed an identity of 97% between vB_EcoM-UFV13 and *Yersinia* virus PST and *Shigella virus* Shfl2, but greater coverage (96%) with the latter.

**Supplementary table-3.** Early, middle and late promoters prediction.

| **Pe**  **(Early promoter)** | **vB_EcoM-UFV13**  **(Genome position)** | **T4**  **(Genome position)** | **Pm**  **(Middle promoter)** | **vB_EcoM-UFV13**  **(Genome position)** | **T4**  **(Genome Position)** | **Pl**  **(Late promoter)** | **vB_EcoM-UFV13**  **(Genome position)** | **T4**  **(Genome position)** |
| --- | --- | --- | --- | --- | --- | --- | --- | --- |
| **1.4..3.9** | 2418..2447 | 2429..2458 | **Pm** | * | * | **soc** | 15434..15451 | 15593..15643 |
| **(3.6)** | Without *mobA* | 2422 | **Pm** | * | * | **Pl** |  |  |
| **(5.9..7.3) = (motB)** | 6684..6738 | 7169..7225 | **Pm(rIIA)** | 2241..2296 | 2252..2307 | **56/69** | Without gp69 | 16803..16856 |
| **(8.1)** | 7797..7826 | 8189..8218 | **Pm(39)** | 4275..4328 | 5339..5392 | **I..TevII** | Without *tevII* | 45621..45671 |
| **(11.5)** | 11661..11690 | 11822..11851 | **Pm(56/69)** | 139406..139429 | 16803..16856 | **49** | 43147.. 43197 | 46875..46925 |
| **12.8 = mod** | 12982..13036 | 13140..13196 | **Pm(61)** | 17800..17853 | 19112..19165 | **rI.1** | 59740 | * |
| **15.0 = soc** | 15434..15451 | 15593..15643 | **Pm(uvsX)** | * | 23742..23796 | **Pe(2)** | 64184..64234 | 67001..67051 |
| **19.8** | 18501..18539 | 20070..20119 | **Pm(segA)** | Without *segA* | 24446..24500 | **Pe(1)** | 64197..64247 | 67014..67064 |
| **20.3 = dmd** | 19590.. 19646 | 20566..20622 | **Pm(42)** | 24339..24392 | 26310..26363 | **Pe(w)** | 64413..64463 | 67230..67280 |
| **26.4 = imm.1** | 25319..25373 | 27034..27090 | **Pm(43)** | 28205..28259 | 29921..29977 | **Pl** | * | 72863 |
| **35.3** | 34167..34216 | 35659..35708 | **Pm(45)** | 31124..31177 | 32616..32669 | **Pl** | * | 74999 |
| **40.4** | 39151..39200 | 41222..41271 | **Pm(45.2)** | 31755..31807 | 33247..33299 | **4** | 75457.. 75507 | 77354..77404 |
| **41.0** | 39596..39645 | 41667..41716 | **Pm(46)** | 33512..33566 | 35004..35058 | **Pl** | * | 77362 |
| **46.7** | 43685..43734 | 47413..47462 | **Pm(47)** | 35070..35127 | 36562..36619 | **Pl** | * | 77381 |
| **50.0 = nrdC.4** | 47297..47353 | 50927..50983 | **Pm(mobB..gt)** | * | * | **53.0** | 75419..75469 | 77316..77366 |
| **54.0** | 50281..50330 | 53904..53953 | **Pm(55)** | 37838..37891 | 40170..40223 | **5.1** | 77856..77906 | 79753..79803 |
| **54.4** | 51720.. 51769 | 55345..55394 | **Pm(55.8)** | 40739.. 40774 | 42795..42849 | **7** | * | * |
| **Pe** | Without mobD.2 | 57954 | **Pm(nrdG)** | * | * | **8** | 83306.. 83356 | 85766..85816 |
| **Pe(57.9)** | 55308.. 55357 | 58741..58790 | **Pm( nrdD+)** | * | * | **9(b)** | 84694..84744 | 87154..87204 |
| **Pe (62.2)** | 59507..59556 | 62758..62807 | **tRNA** | 70970..71020 | 72859..72909 | **9(a)** | 84254..84304 | 86714..86764 |
| **64.6 = denV** | 67498..67541 | 65375..65423 | **57A** | 72983..73035 | 74867..74919 | **10** | 85379..85429 | 87839..87889 |
| **Pe (65.0)** | Without *IpII* | 65760..65809 | **1** | 73486..73539 | 75382..75435 | **15** | 92832..92882 | 95291..95341 |
| **Pe** |  | 66462 | **uvsY** | 111580..111634 | 115361..115415 | **17** | 93648..93698 | 96107..96157 |
| **69.9** | 67116.. 67155 | 69928..69977 | **Pm(30)** | * | * | **18(w)** | 96008..96058 | 98467..98517 |
| **69.4** | 71640..71677 | 70320..70369 | **Pm(30.2)** | * | * | **Pl** | * | * |
| **69.8** | 68057..68106 | 70657..70706 | **Pm(31)** | * | * | **P19(a)** | 98059..98109 | 100518..100568 |
| **tRNA.4 = 72.6** | 71633..71687 | 73526..73582 | **Pm(l..tevIII)** | * | * | **P19(b)** | 98118..98168 | 100577..100627 |
| **73.0** | 72004..72053 | 73900..73949 | **Pm(nrdB)** | * | 139868..139921 | **20** | 98679..98729 | 101138..101188 |
| **Pe** | * | 79405 | **Pm(nrdA)** | 139453..139508 | 142714..142769 | **67** | 100034..100084 | 102493..102543 |
| **alt.3** | F:119252..119300 | 123057 | **Pm(td+)** | 141272..141326 | 145131..145185 | **21** | 100638..100688 | 103100..103150 |
| **128.2** | 127666..127714 | 129880..129928 | **Pm(32)** | 143143..143192 | 148054..148101 | **22** | 101587..101637 | 104049..104099 |
| **128.6** | 128057..128106 | 130271..130320 | **Pm(dsbA)** | 145492..145547 | 149861..149916 | **Pl** | * | * |
| **131.7** | 130952..131001 | 133292..133341 | **Pm(34)** | 146365..146415 | 150734..150784 | **23** | 102309..102358 | 104770..104820 |
| **134.4** | 134276..134325 | 136297..136346 |  |  |  | **23(w)** | 102279..102329 | 104741..104791 |
| **144.6** | 143055..143104 | 146836..146885 |  |  |  | **hoc.1** | 108867..108886 | 112025..112075 |
| **148.6** | 146355..146404 | 150724..150773 |  |  |  | **hoc.2** | 108830..108880 | 111988..112038 |
| **158.7** | 157832.. 157881 | 161172..161221 |  |  |  | **Pl** | * | * |
| **161.1 = motA** | 160290.. 160346 | 163627..163683 |  |  |  | **Pl(segE)** | * | * |
| **164.2** | 163437..163484 | 166768..166817 |  |  |  | **PluvsY...2** | * | * |
| **164.5** | 163924..163973 | 167047..167096 |  |  |  | **Pl** | * | * |
|  |  |  |  |  |  | **P26(a)** | 112659..112709 | 116440..116490 |
|  |  |  |  |  |  | **P26(w)** | 112650..112700 | 116431..116481 |
|  |  |  |  |  |  | **Pl** |  |  |
|  |  |  |  |  |  | **Pl(alt)** | 123490..123534 | 125521..125571 |
|  |  |  |  |  |  | **Pl(rIII)** | * | * |
|  |  |  |  |  |  | **Pl(alc)** | * |  |
|  |  |  |  |  |  | **Pl(I..TevIII)** | * | * |
|  |  |  |  |  |  | **Pl(I..TevI)** | * | 144445..144495 |
|  |  |  |  |  |  | **Pl(32)** | * | 147994..148044 |
|  |  |  |  |  |  | **Pl(rnh)** | * |  |
|  |  |  |  |  |  | **Pl35** | * |  |
|  |  |  |  |  |  | **Pl36** | 151432..151482 | 155804..155854 |
|  |  |  |  |  |  | **Pl37** | 151948..151998 | 156323..156373 |
|  |  |  |  |  |  | **Pl38** | * |  |
|  |  |  |  |  |  |  |  |  |


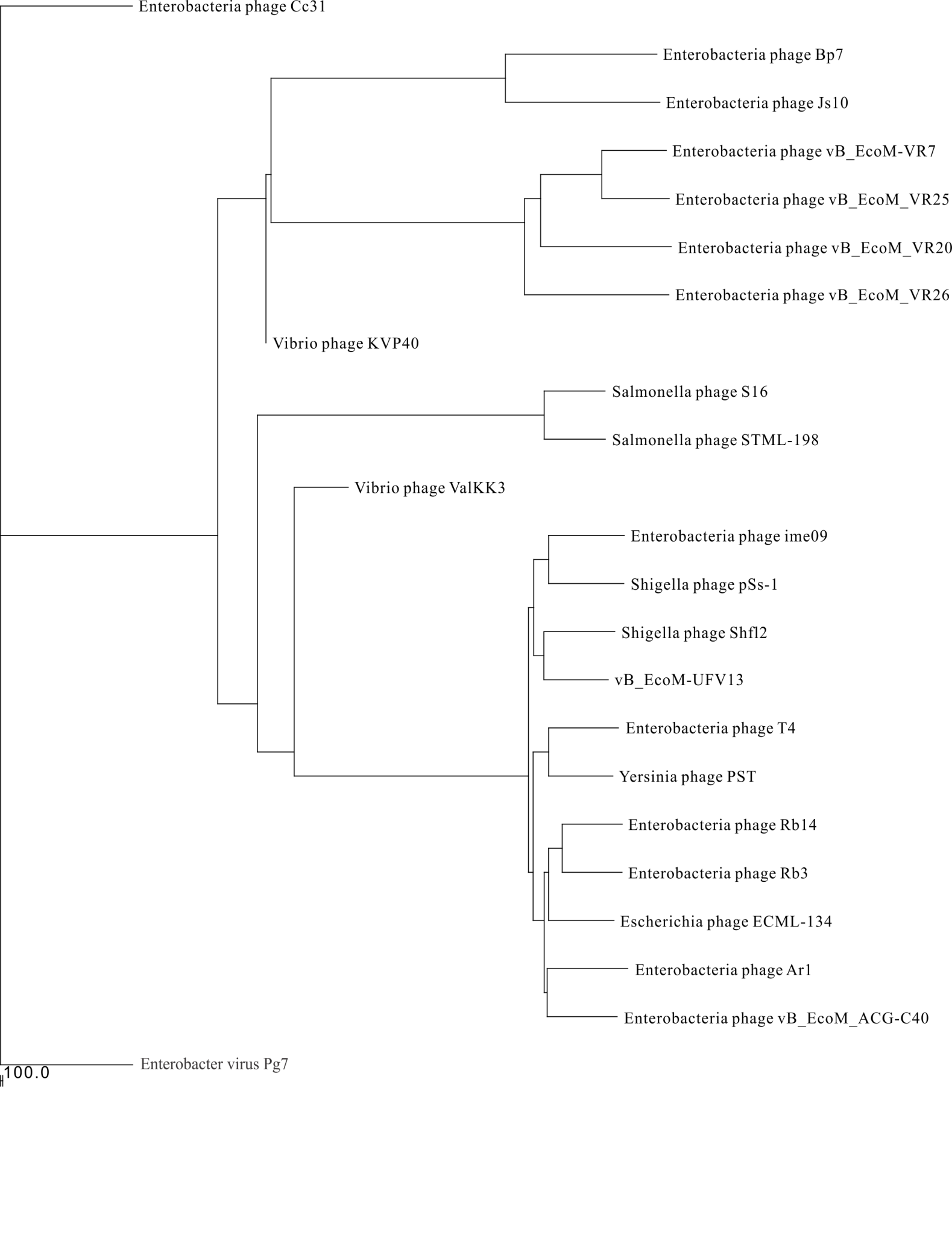
Supplementary figure-2. SNP-based phylogentic tree supports the phylogenetic relationship between UFV13 and *Shigella* phage Shfl2.


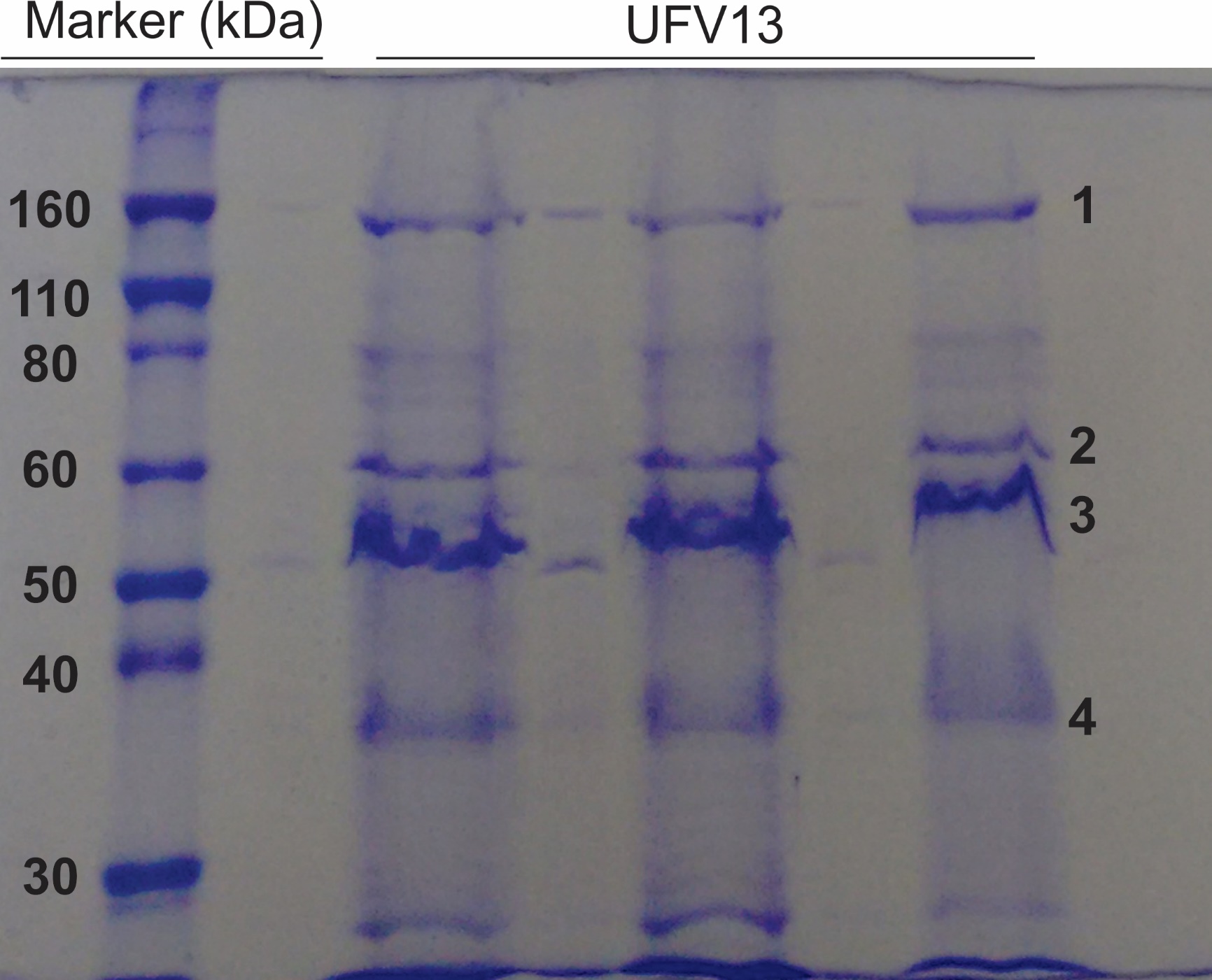
**Supplementary figure-3.** *Escherichia phage* UFV13 protein profile by SDS-PAGE. MALDI/TOF-TOF analyze revealed the presence of host receptor protein after virus purification. Sample 1 – vB_EcoM-UFV13 long tail fiber proximal subunit); Sample 2 – *E. coli* chaperonin GroL; Sample 3 – *Escherichia* phage major capsid protein; Sample 4 – *E. coli* outer membrane protein C (OmpC). Three lanes were loaded with the aim to increase protein yield for MALDI/TOF-TOF analyze.

**Supplementary table-4.** NCBI accession number list of all viral genomes used in this study.

| Virus | Accession number |
| --- | --- |
| *Escherichia virus RB3* | KM606994 |
| *Escherichia virus RB14* | NC_012638 |
| *Escherichia virus ECML134* | JX128259 |
| *Escherichia virus C40* | JN986846 |
| *Escherichia virus AR1* | AP011113 |
| *Escherichia virus T4* | AF158101.6 |
| *Yersinia virus PST* | KF208315 |
| *Escherichia virus UFV13* | NC_031103 |
| *Shigella virus Shfl2* | HM035025 |
| *Escherichia virus ime09* | JN202312 |
| *Shigella virus Pss1* | KM501444 |
| *Enterobacter virus CC31* | NC_014662 |
| *Enterobacter virus PG7* | NC_023561 |
| *Salmonella virus S16* | NC_020416 |
| *Salmonella virus STML198* | JX181825 |
| *Enterobacter virus JS10* | NC_012741 |
| *Enterobacter virus VR26* | NC_028957 |
| *Enterobacter virus VR20* | NC_028894 |
| *Enterobacter virus VR7* | NC_014792 |
| *Enterobacter virus VR25* | NC_028925 |
| *Bacillus virus BMBtp1* | KT852578 |

**Supplementary figure-4.** Experimental design of *E. coli*-induced mastitis in mouse
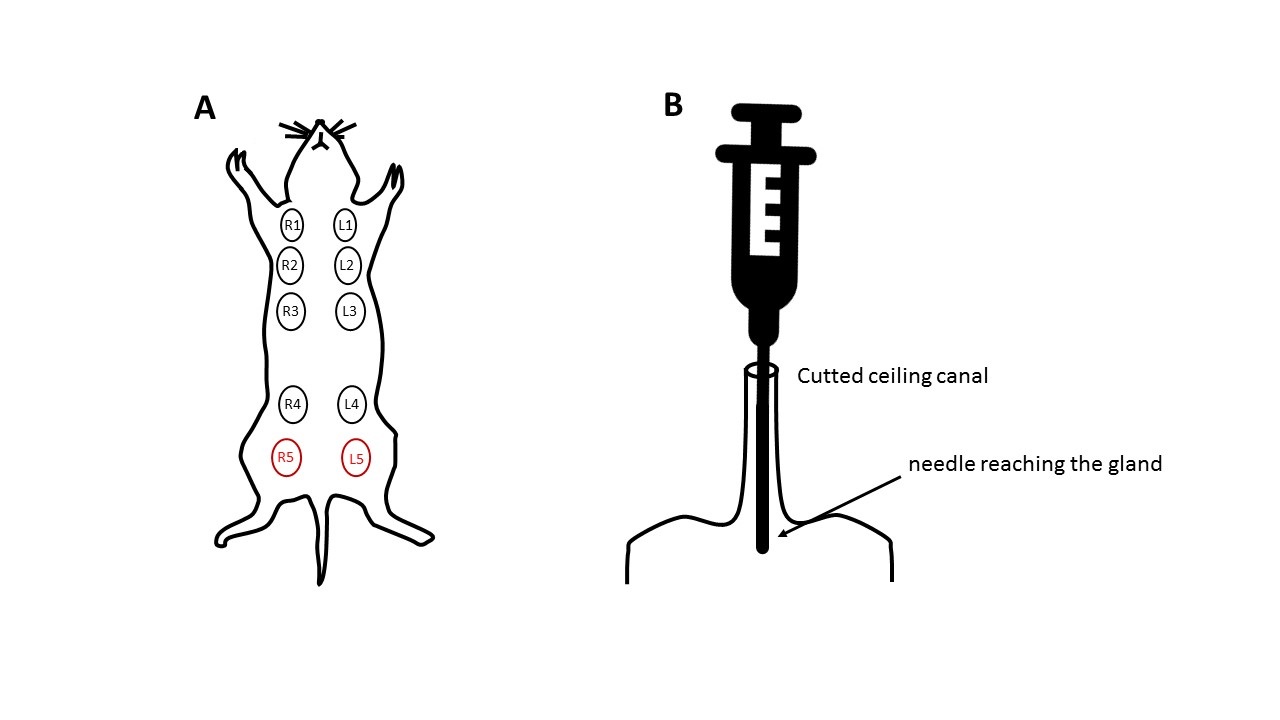
. A – The last two abdominal ceilings (R5 and L5) of each animal were used. B – After that the lactating mouse was intraperitoneally anesthetized, R5 and L5 were assessed by cutting the teat canal.

**Supplementary Figure-5.** A 10-fold reduction of *E. coli 30* load was observed after viral inoculation using MOI 10.

**
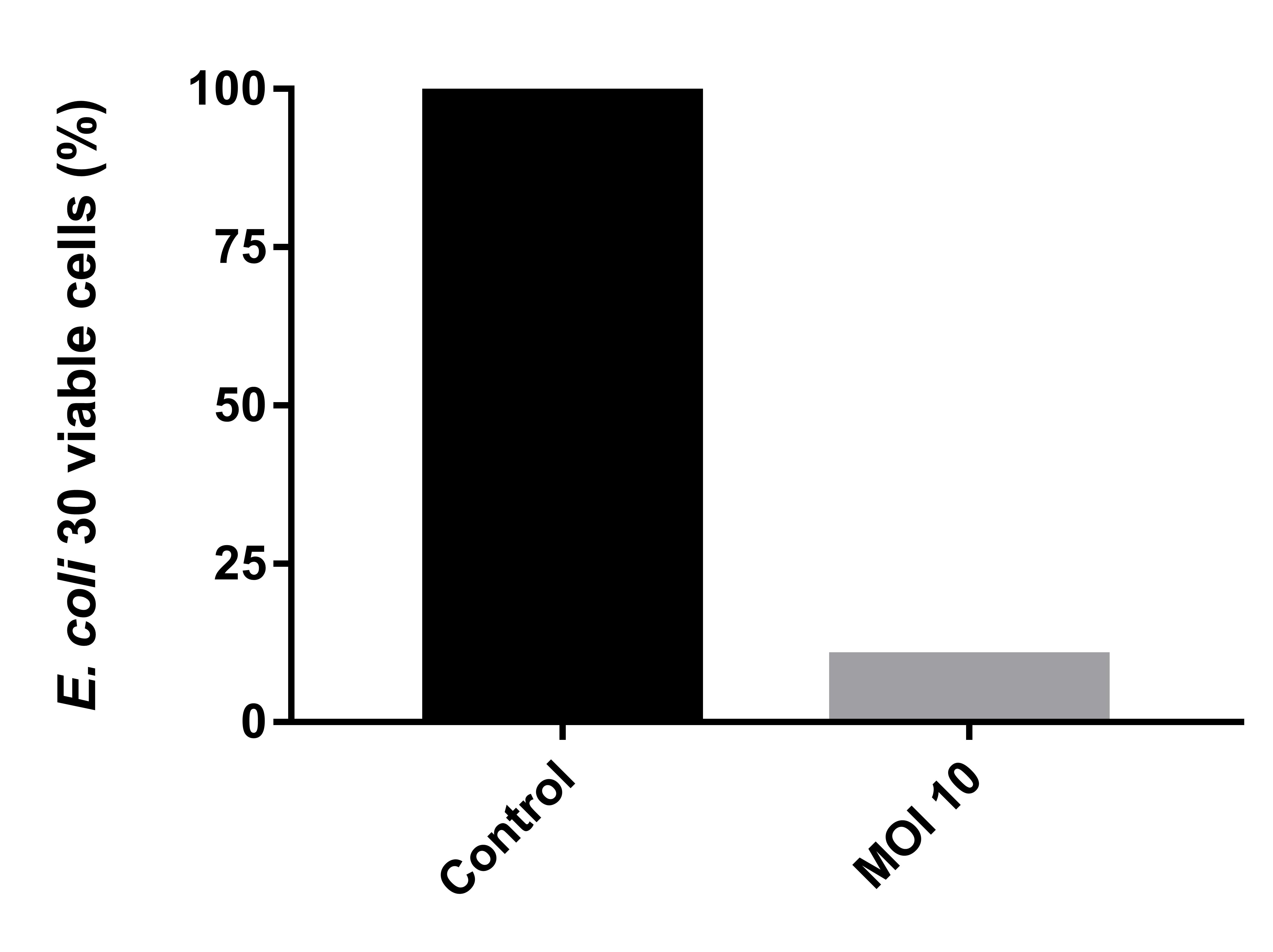
**

**Supplementary Table-5. Antimicrobial susceptibility test was performed by disc diffusion assay using 25 different antibiotics. *E. coli* 30 was resistant to 56% of antibiotics tested.**

| **Antibiotics** | **Code** | **Resistant** | **Intermediate** | **Susceptible** | **Diameter (cm)** | **Result** |
| --- | --- | --- | --- | --- | --- | --- |
| Ceftriaxone | CRO 30 | ≤19 | 20-22 | ≥23 | 19 | Resistant |
| Bacitracin | BAC 10 | - | - | - | 0.6 | Resistant |
| Amoxicillin | AMO 10 | - | - | - | 0.6 | Resistant |
| Cefalotin | CFL 30 | ≤14 | 15-17 | ≥18 | 13 | Resistant |
| Linezolid | LNZ 30 | ≤20 | 21-22 | ≥23 | 0.6 | Resistant |
| Sulfamethoxazole/Trimethoprim | SUT 25 | ≤10 | 11-15 | ≥16 | 0.6 | Resistant |
| Tetracyclin | TET 30 | ≤11 | 12-14 | ≥15 | 0.6 | Resistant |
| Penicillin G | PEN 10 | ≤28 | - | ≥15 | 0.6 | Resistant |
| Erythromycin | ERI 15 | ≤15 | 16-20 | ≥21 | 0.6 | Resistant |
| Oxacillin | OXA 01 | - | - | - | 0.6 | Resistant |
| Ampicillin | AMP 10 | ≤13 | 14-16 | ≥17 | 0.6 | Resistant |
| Clindamycin | CLI 02 | ≤15 | 16-18 | ≥21 | 0.6 | Resistant |
| Vancomycin | VAN 30 | ≤14 | 15-16 | ≥17 | 0.6 | Resistant |
| Rifampicin | RIF 05 | ≤16 | 17-18 | ≥19 | 0.6 | Resistant |
| Chloramphenicol | CLO 30 | ≤12 | 13-17 | ≥18 | 25 | Susceptible |
| Ceftazidime | CAZ 30 | ≤17 | 18-20 | ≥21 | 28 | Susceptible |
| Aztreonam | ATM 30 | ≤17 | 18-20 | ≥21 | 32 | Susceptible |
| Amikacin | AMI 30 | ≤14 | 15-16 | ≥17 | 28 | Susceptible |
| Amoxicillin/Clavulanate | AMC 30 | ≤13 | 14-17 | ≥18 | 20 | Susceptible |
| Cefepime | CPM 30 | ≤18 | - | ≥21 | 35 | Susceptible |
| Ciprofloxacin | CIP 05 | ≤15 | 16-20 | ≥21 | 29 | Susceptible |
| Azithromycin | AZI 15 | ≤13 | 14-17 | ≥18 | 27 | Susceptible |
| Cefoxitin | CFO 30 | ≤14 | 15-17 | ≥18 | 32 | Susceptible |
| Gentamicin | GEN 10 | ≤12 | 13-14 | ≥15 | 22 | Susceptible |
| Piperacillin/Tazobactam | PIT 110 | ≤17 | 18-20 | ≥21 | 23 | Susceptible |

**Supplementary Table-6.** Biofilm formation and motility assays. *E. coli* 30 displays the capability to form biofilm and possesses swarming and swimming abilities.

| **Virulence factors** | **Result** |
| --- | --- |
| Swarming | + |
| Swimming | + |
| Twitching | - |
| Biofilm | + |
